# Establishment of human iPSC-based models for the study and targeting of glioma initiating cells

**DOI:** 10.1038/ncomms10743

**Published:** 2016-02-22

**Authors:** Ignacio Sancho-Martinez, Emmanuel Nivet, Yun Xia, Tomoaki Hishida, Aitor Aguirre, Alejandro Ocampo, Li Ma, Robert Morey, Marie N. Krause, Andreas Zembrzycki, Olaf Ansorge, Eric Vazquez-Ferrer, Ilir Dubova, Pradeep Reddy, David Lam, Yuriko Hishida, Min-Zu Wu, Concepcion Rodriguez Esteban, Dennis O'Leary, Geoffrey M. Wahl, Inder M. Verma, Louise C. Laurent, Juan Carlos Izpisua Belmonte

**Affiliations:** 1 Gene Expression Laboratory Belmonte, Salk Institute for Biological Studies, 10010 North Torrey Pines Road, La Jolla, California 92037, USA; 2 Universidad Católica San Antonio de Murcia (UCAM) Campus de los Jerónimos, N° 135 Guadalupe, Murcia 30107, Spain; 3 Department of Reproductive Medicine, University of California, San Diego, Sanford Consortium for Regenerative Medicine, 2880 Torrey Pines Scenic Drive, La Jolla, California 92037, USA; 4 Molecular Neurobiology Laboratory, Salk Institute for Biological Studies, 10010 North Torrey Pines Road, La Jolla, California 92037, USA; 5 Department of Neuropathology, West Wing, Level 1, John Radcliffe Hospital, Oxford OX3 9DU, UK; 6 Gene Expression Laboratory Wahl, Salk Institute for Biological Studies, 10010 North Torrey Pines Road, La Jolla, California 92037, USA; 7 Laboratory of Genetics, Salk Institute for Biological Studies, 10010 North Torrey Pines Road, La Jolla, California 92037, USA

## Abstract

Glioma tumour-initiating cells (GTICs) can originate upon the transformation of neural progenitor cells (NPCs). Studies on GTICs have focused on primary tumours from which GTICs could be isolated and the use of human embryonic material. Recently, the somatic genomic landscape of human gliomas has been reported. RTK (receptor tyrosine kinase) and p53 signalling were found dysregulated in ∼90% and 86% of all primary tumours analysed, respectively. Here we report on the use of human-induced pluripotent stem cells (hiPSCs) for modelling gliomagenesis. Dysregulation of RTK and p53 signalling in hiPSC-derived NPCs (iNPCs) recapitulates GTIC properties *in vitro*. *In vivo* transplantation of transformed iNPCs leads to highly aggressive tumours containing undifferentiated stem cells and their differentiated derivatives. Metabolic modulation compromises GTIC viability. Last, screening of 101 anti-cancer compounds identifies three molecules specifically targeting transformed iNPCs and primary GTICs. Together, our results highlight the potential of hiPSCs for studying human tumourigenesis.

## Abstract (web summary)

Glioma can originate from the transformation of neural progenitor cells into glioma initiating cells. Here, the authors demonstrate the use of induced pluripotent stem cells as a suitable model for generating neural progenitor cells, which can be subsequently transformed to glioma initiating cells that are able to the generate human glioma-like tumours in mice.

## Graphical abstract

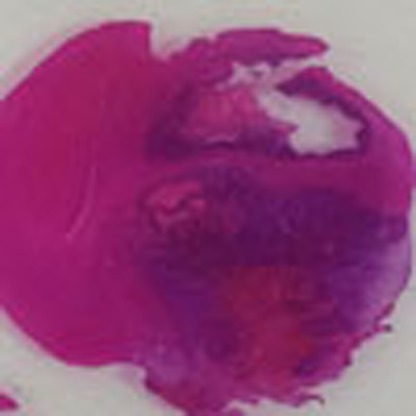

Adult gliomas are the most malignant human brain tumours^1^, with no curative therapy available. Gliomas can originate as a result of adult NPCs transformation to glioma tumour-initiating cells (GTICs)^1^,^2^. However, strategies for studying the mechanisms underlying the transformation of adult human NPCs to GTICs remain scarce with most mechanistic studies relying on the use of transgenic murine models^1^. Recent reports have highlighted the potential of reprogramming to induce the conversion of differentiated glioma cells to a GTIC-like phenotype^3^. Despite much success, reprogramming of cancer cells to GTICs requires the use of already transformed cells isolated from a pre-existent tumour^1^,^3^,^4^,^5^,^6^,^7^,^8^,^9^,^10^. Thus, and similar to the use of primary glioma cells, such reprogrammed GTICs prevent functional studies on the mechanisms leading to NPC transformation and tumour initiation. Accordingly, functional studies on NPC transformation and GTIC formation remain largely limited to the use of differentiated neural derivatives^11^,^12^ and/or the use of murine models^1^,^13^,^14^. Contrary to murine models, isolation of adult human NPCs remains restricted to brain tissue material obtained from patients with pathological conditions, such as epilepsy, or post-mortem. As an alternative to study human gliomagenesis, the use of primary fetal NPCs and NPCs differentiated from human embryonic stem cells has been reported^1^,^2^,^10^,^15^,^16^. However, the use of embryonic/fetal material remains the subject of ethical controversy and limits the possibility for investigating the role of different mutations in various genetic backgrounds represented in the human population.

In 2006, Kazutoshi Takahashi and Shinya Yamanaka were able to reprogram somatic cells into pluripotent stem cells upon the forced expression of a small number of defined genes^17^. Reprogramming to human-induced pluripotent stem cells (hiPSCs) possesses the inherent advantages of voiding the need for embryonic material while allowing for the generation of pluripotent cells from any given genetic background in a patient-specific manner. The possibility for generating patient-specific iPSCs holds great promise for the future development of autologous cell therapies as well as open unprecedented opportunities for disease modelling and drug discovery studies^18^. In addition, modelling of complex phenotypes, such as aging, can be accomplished by overexpressing specific mutant genes in otherwise wild-type hiPSCs^19^,^20^. Therefore, the use of hiPSCs, and/or their derivatives, in which defined genetic alterations related to cancer are introduced might represent a suitable strategy for the establishment of human cancer models.

Here we report on the establishment of tractable *in vitro* and *in vivo* hiPSC models for the study of human iNPC transformation to GTIC-like cells. Genetic manipulation of p53 and receptor tyrosine kinase signalling leads to the acquisition of cancer stem cell-like features *in vitro*, including enhanced self-renewal and migratory properties alongside metabolic reprogramming. Transformation of iNPCs to GTIC-like cells leads to the generation of human glioma-like tumours with distinct histopathological features upon orthotopic transplantation of 500 cells into the murine brain as well as in serial transplantation experiments. Ultimately, our results highlight the suitability of human iPSC technologies for the modelling of human cancer.

## Results

### Transformation of human iPSC-derived neural progenitors

Mutations affecting components of the p53 signalling pathway are collectively observed in ∼86% of all human gliomas^21^. Therefore, we first generated hiPSC lines from healthy/‘wild-type' human fibroblasts (hereafter referred to as _WT_iPSCs) and lines in where p53 was knocked down (hereafter referred to as _p53KD_iPSCs) (Supplementary Fig. 1a). All hiPSCs presented the typical hallmarks of pluripotency including *in vivo* teratoma formation in the absence of apparent malignant transformation (Supplementary Fig. 1b–f). Next, we differentiated NPCs from the generated hiPSCs (Supplementary Fig. 2a). Immunofluorescence analysis as well as multilineage differentiation potential confirmed the NPC identity of the differentiated cells (hereafter referred to as iNPCs) (Supplementary Fig. 2b–d). We have previously reported that human glioma infiltration is driven by activation of Src-family kinases (SFKs) and targeting SFKs has emerged as an attractive therapeutic approach currently under development^20^,^21^,^22^,^23^. In addition, Brennan *et al*.^21^ described mutations collectively leading to PI3K and MAPK hyperactivation in ∼90% of human gliomas. Therefore, we additionally generated iNPCs-overexpressing mutant-active versions of Src (to mimic infiltrative behaviour), EGFR (duplicated and/or mutated in ∼57% of human gliomas)^21^ and Ras (mutated in ∼1% of human gliomas and a common upstream regulator of both PI3K and MAPK signalling)^21^. Lentivirus-mediated overexpression of the different mutant genes in either 293T cells or _WT-_ and _p53KD-_ iNPCs (namely _Ras/EGFR/Src_iNPCs and _p53KD-Ras/EGFR/Src_iNPCs, respectively) confirmed the hyperactivation of PI3K and MAPK pathways as demonstrated by increased AKT and ERK phosphorylation, respectively (Supplementary Fig. 2a,e,f).

**Figure 1:**
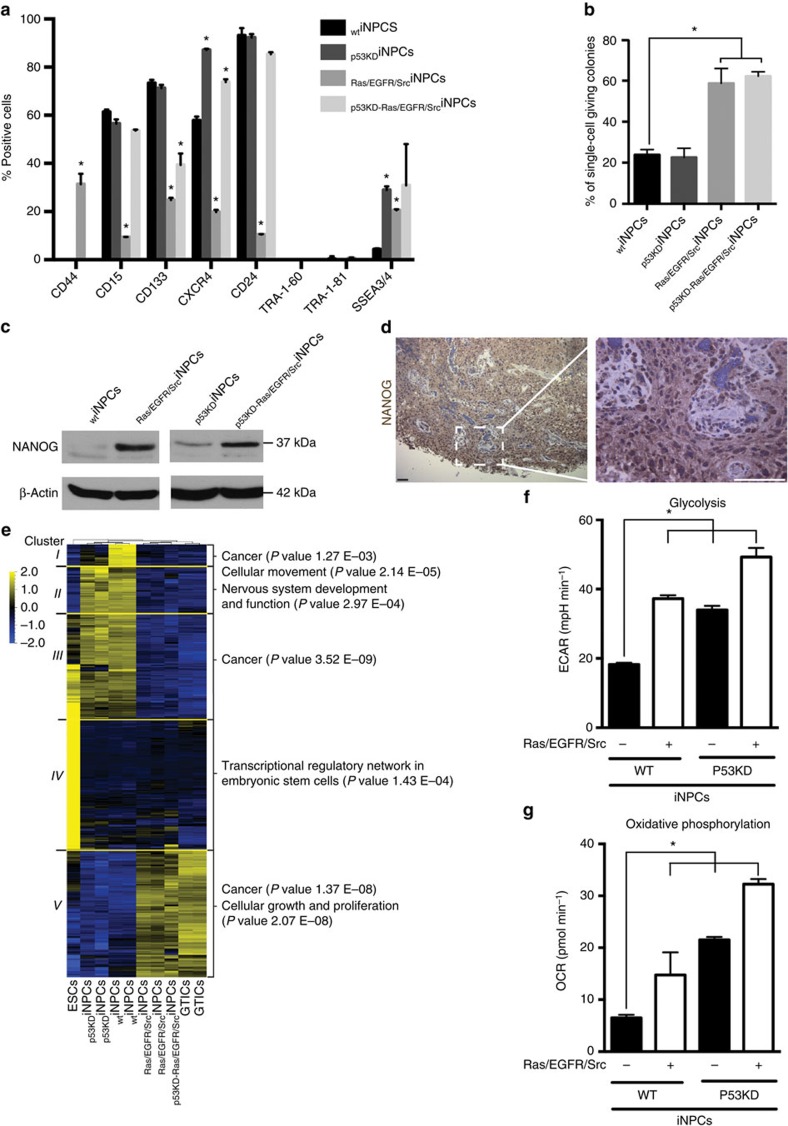
Transformation of human iNPCs results in the acquisition of a GTIC-like phenotype *in vitro*. (**a**) Flow cytometry analysis for the indicated markers in different transformed and wild-type iNPC groups (*n*=4/group with technical duplicates). (**b**) Transformation of human iNPCs leads to increased self-renewal properties only in cells where PI3K and MAPK signalling is dysregulated by overexpression of *Ras/EGFR/Src* mutant genes as highlighted by single-cell self-renewal assays (*n*=3/group with 24 technical replicates). (**c**) iNPC transformation results in the upregulation of endogenous NANOG as highlighted by western blot analyses. (**d**) NANOG expression is upregulated in primary human adult glioma samples. (**e**) Unsupervised cluster analysis of mRNA expression data highlighting the similarities between transformed iNPCs and primary GTICs. Please note that _p53KD_iNPCs are more similar to _WT_iNPCs than to primary GTICs. (**f**,**g**) Transformation of human iNPCs induces glycolytic (**f**) and oxidative phosphorylation changes as indicated by Seahorse analysis (**g**), as measured by ECAR and OCR (*n*=4/group with four technical replicates). Data are represented as mean ±s.d. *P* values were calculated by Student's *t*-test or Mann–Whitney test when appropriate and represented as follows: *P<0.05. Scale bars, 200 μm (**d**).

GTICs were first isolated based on the expression of CD133 and thus CD133+ thought to unequivocally label glioma cells with stem cell properties^26^. However, CD133− glioma cells presenting stem cell and GTIC properties have also been reported^27^,^28^ and CD133+ cells found in a variety of normal tissues other than tumours^29^. Nevertheless, we sought to investigate the role of putative GTIC surface markers for enriching cancer populations with stem cells properties. On the basis of our flow cytometry results (Fig. 1a), we first separated CD133+ and CD133− cells and performed *in vitro* single-cell tumour forming assays. To avoid limiting our analyses to a single marker, we also sorted out CD15+ and CD15− cells as well as CXCR4+ and CXCR4− populations. All different cell populations demonstrated comparable colony forming potential (Supplementary Fig. 2g). These results are in agreement with the notion that GTICs are very heterogeneous. Indeed, a universally accepted panel of markers for the characterization and isolation of GTICs is yet to be reported^30^. Variability in surface marker expression in cancer cells bearing stem cell properties is not exclusive to gliomas and has now been observed in a variety of human tumours^1^,^3^,^4^,^5^,^6^,^7^,^9^,^10^. Because of surface marker heterogeneity and their expression in certain non-transformed adult stem cells, recent reports advocate for the characterization of cancer stem cells based on functional properties, such as multilineage differentiation potential and tumour formation upon serial transplantation^1^,^3^,^4^,^5^,^6^,^7^,^9^,^10^. Accordingly, we next decided to investigate the self-renewal potential of non-sorted iNPCs by performing single-cell assays. Enhanced self-renewal properties were observed in all iNPCs where PI3K/MAPK signalling was dysregulated (Fig. 1b). Furthermore, NANOG, a protein expressed in iPSCs, was found upregulated in transformed iNPCs (Fig. 1c). Interestingly, immunohistochemical analyses of human brain tumour samples further confirmed NANOG expression in grade IV gliomas (Fig. 1d). Lack of TRA1-60 and TRA1-81 expression (Fig. 1a) further demonstrated that NANOG expression was due to transformation and not the presence of undifferentiated hiPSCs.

### Transformation of human NPCs generates GTIC-like cells

We next compared the generated iNPCs with primary GTICs, isolated and characterized by Pollard *et al*.^5^, by performing genome-wide analyses. Genome-wide expression data demonstrated that transformed iNPCs closely resemble primary GTICs at the molecular level, though some differences were readily observed among the different groups. _WT_iNPCs were most similar to _p53KD_iNPCs, while _Ras/EGFR/Src_iNPCs and _p53KD-Ras/EGFR/Src_iNPCs were very similar to each other and intermediate between the _WT_iNPC/_p53KD_iNPC cluster and primary GTICs (Supplementary Data 1, analysis of the variance (ANOVA) *q* value <0.01 and variance filter=0.2 using Qlucore). The corresponding heatmap showed five large clusters of genes that were differentially expressed among _WT_iNPCs, _p53KD_iNPCs, _Ras/EGFR/Src_iNPCs, _p53KD-Ras/EGFR/Src_iNPCs and GTICs (Fig. 1e, clusters I–V and Supplementary Data 2). Importantly, genes in cluster V showed higher expression in the GTICs, _Ras/EGFR/Src_iNPCs and _p53KD-Ras/EGFR/Src_iNPCs compared with ESCs, _WT_iNPCs, and _p53KD_iNPCs (Fig. 1e, cluster V and Supplementary Data 2). Of the five clusters, cluster V showed the highest number of significant enrichments on pathways analysis, and was enriched for several cell growth-, proliferation- and cancer-associated pathways. Notably, DNA methylation analysis confirmed a more undifferentiated phenotype for _Ras/EGFR/Src_iNPCs and _p53KD-Ras/EGFR/Src_iNPCs (Supplementary Table 1).

Changes in the energetic metabolism are generally observed in cancer cells, the so-called Warburg effect^31^. In agreement, Seahorse analyses demonstrated the clear metabolic reprogramming of transformed iNPCs (Fig. 1f,g). RNA expression analyses highlighted the upregulation of glycolytic genes typically associated with metabolic alterations in GTICs in the transformed iNPCs, including: *PKM2*; *HK*; *GLUT3* (a gene recently described as critical for glucose uptake in GTICs)^6^; and *LDHB* (a gene commonly upregulated in gliomas bearing IDH mutations)^32^ (Supplementary Fig. 3a). Higher lactate production and lower glucose consumption in transformed iNPCs revealed significant differences in the lactate/glucose ratio (Supplementary Fig. 3b). Non-labelled mass spectrometry highlighted, as before, higher similarities between _p53KD_- and _WT_iNPCs, an intermediate profile in _Ras/EGFR/Src_iNPCs and marked differences in the levels of metabolites involved in carbon and aminoacid metabolism in _p53KD-Ras/EGFR/Src_iNPCs (Supplementary Fig. 3c). Next, U-13C glucose labelling was used to monitor carbon flux in the transformed iNPCs as compared with _WT_iNPCs. No significant differences in levels of production of glycolytic metabolites were found among the four lines, except for increased glucose flux into lactate and glycine in _p53KD-Ras/EGFR/Src_iNPCs, (Supplementary Fig. 3d). Interestingly, we observed much lower influx of glucose into glutamate production and into the trichloroacetic acid or tricarboxylic acid cycle (as highlighted by differential labelling of aspartate, malate, fumarate and α-ketoglutarate) in _Ras/EGFR/Src_iNPCs and _p53KD-Ras/EGFR/Src_iNPCs, compared with _WT_- and _p53KD_iNPCs, which shared a similar metabolic profile (Supplementary Fig. 3d). Quantification of ROS levels further highlighted metabolic reprogramming and demonstrated reduced mitochondrial ROS formation upon iNPC transformation, whereas total ROS levels remained comparable across all different groups (Supplementary Fig. 4a,b). MitoTracker analysis indicated that disruption of signalling pathways resulted in morphological mitochondrial changes and higher fragmentation of mitochondrial networks as compared with _WT_iNPCs (Supplementary Fig. 4c).

**Figure 2:**
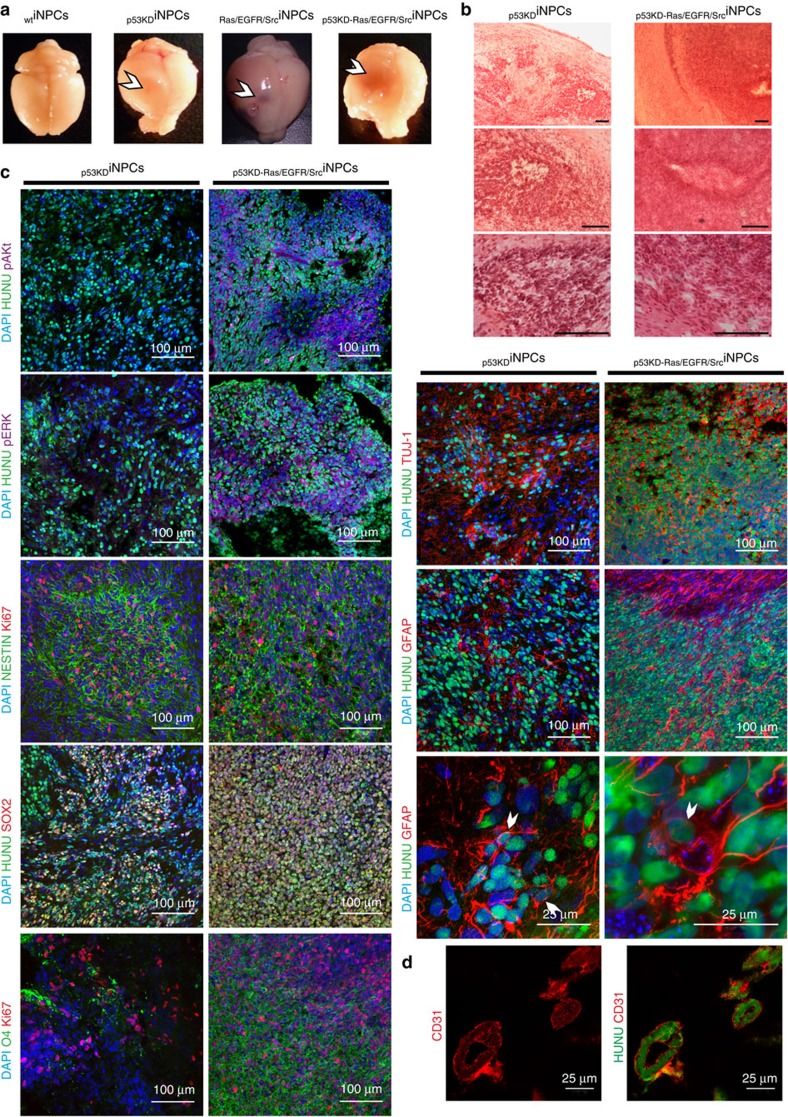
Transformation of human iNPCs results in the acquisition of GTIC-like properties *in vivo*. (**a**) Representative pictures demonstrating the formation of vascularized tumours only in animals receiving transformed, and not wild-type, human iNPCs. (**b**) Haematoxylin-eosin staining demonstrating the presence of highly aggressive brain tumours upon orthotopic transplantation of transformed human iNPCs into the murine brain. (**c**) Immunofluorescence analysis demonstrating the presence of undifferentiated SOX2+ cells (red) as well as differentiation into the three major neural lineages upon transplantation of transformed human iNPCs. Please note the higher cellularity in tumours derived upon transplantation of _p53KD-Ras/EGFR/Src_iNPCs. HUNU (green) indicates human nuclear antigen staining; O4 (green) indicates oligodendrocyte differentiation; Tuj1 (red) indicates neuronal differentiation; and GFAP (red) indicates glial differentiation. pERK (purple) and pAKT (purple) were only detected in tumours generated upon transplantation of iNPCs-overexpressing mutant versions of *Ras/EGFR/Src*. Cells were counterstained with DAPI (blue) and their proliferative state monitored by Ki67 immunostaining (red). (**d**) Brain tumours derived from human _p53KD-Ras/EGFR/Src_iNPCs demonstrated the presence of human vessels derived from the injected GTIC-like cells as indicated by CD31 and HUNU co-localization. *n*=5 animals/condition. A minimum of three brain sections/animal/condition were analysed. Scale bars, 200 μm (**b**); 100 μm or 25 μm as indicated (**c**); and 25 μm (**d**).

To test whether transformation of iNPCs resulted in the functional acquisition of GTIC-like properties *in vivo*^4^,^7^,^9^, we performed xenograft transplantation studies. Orthotopic injection of transformed iNPCs (500,000 cells) into the murine brain resulted in the rapid appearance of highly aggressive tumours (Fig. 2a and Supplementary Table 2). Tumours were then analysed for the presence of classical hallmarks of human glioblastoma multiforme (GBM) such as pleomorphic GFAP+ glial cells, mitoses, diffuse infiltrative growth, pseudopalisading microscopic necrosis and microvascular proliferation (Fig. 2b–d and Supplementary Fig. 5a,b). Immunofluorescence analysis demonstrated the presence of de-differentiated NESTIN+ and SOX2+ cancer cell populations as well as oligodendrocytes (O4), astrocytes (GFAP) and neurons (TUJ-1) confirming the *in vivo* differentiation potential of the generated GTIC-like cells (Fig. 2c and Supplementary Fig. 5b). In addition to the presence of undifferentiated tumour cells, all tumours presented mitotic figures. Thus, these data suggested a pathological classification as high-grade astrocytomas (grades III–IV). In line with the differences observed *in vitro*, *in vivo* analyses highlighted intergroup differences reflecting different glioma subtypes. Tumours arising from _p53KD_iNPCs were reminiscent of ‘small-cell' GBM, showing elongated spindle cells with eosinophilic cytoplasm alternating with clusters of compact cells with little cytoplasm and a high nuclear:cytoplasmic ratio. These tumours displayed a diffuse growth pattern but lacked the pleomorphism usually seen in GBM and no obvious transition zone to normal brain was detected. Brain tumours developed upon injection of _Ras/EGFR/Src_iNPCs showed a nodular growth with perivascular spread but not much diffuse parenchymal invasion was observed. Interestingly, some necrosis was observed but no pseudopalisading. Notably, the pale cytoplasm and elongated shape that were observed are reminiscent of mesenchymal morphology. Lastly, from all three groups analysed, the tumours developed after injection of _p53KD-Ras/EGFR/Src_iNPCs closely resembled grade IV glioblastomas. These tumours demonstrated the highest cellularity and massive necrotic areas as well as microvascularization, pseudopalisading, breaching of the pial surface and invasion of the subarachnoid space as seen in GBM and gliosarcomas. Most notably, transdifferentiated human CD31+ endothelial cells a feature of human gliomas for promoting tumour vascularization^33^, were only observed upon injection of _p53KD-Ras/EGFR/Src_iNPCs (Fig. 2d). Animals injected with _WT_iNPCs lived their normal lifespan and did not present brain tumours (Supplementary Table 2) whereas injection of undifferentiated iPSCs led to the formation of well-defined embryonic-like teratomas histologically distinct from the brain tumours generated upon transplantation of transformed iNPCs (Supplementary Fig. 1e). Next, we performed xenograft transplantation experiments with limited cell numbers as well as secondary tumour formation assays. Among the transformed groups, only iNPCs dysregulated for PI3K and MAPK signalling (_Ras/EGFR/Src_iNPCs and _p53KD_-_Ras/EGFR/Src_iNPCs) were able to form primary tumours when 500 cells were orthotopically injected into the murine brain (Supplementary Fig. 6a and Supplementary Table 3). Furthermore, serial transplantation of _Ras/EGFR/Src_iNPCs and _p53KD_-_Ras/EGFR/Src_iNPCs resulted in the formation of highly aggressive and infiltrative secondary tumours, presenting similar histological characteristics to those observed in the respective primary tumours, even upon secondary transplantation of 500 cells (Supplementary Fig. 6b–e and Supplementary Table 3). In agreement with the similarities between _p53KD_iNPCs and _WT_iNPCs, _p53KD_iNPCs did not lead to secondary tumour formation upon serial transplantation regardless of the number of cells injected (Supplementary Table 3).

### Transformation of iNPCs results in differential SOX2 binding

**Figure 3:**
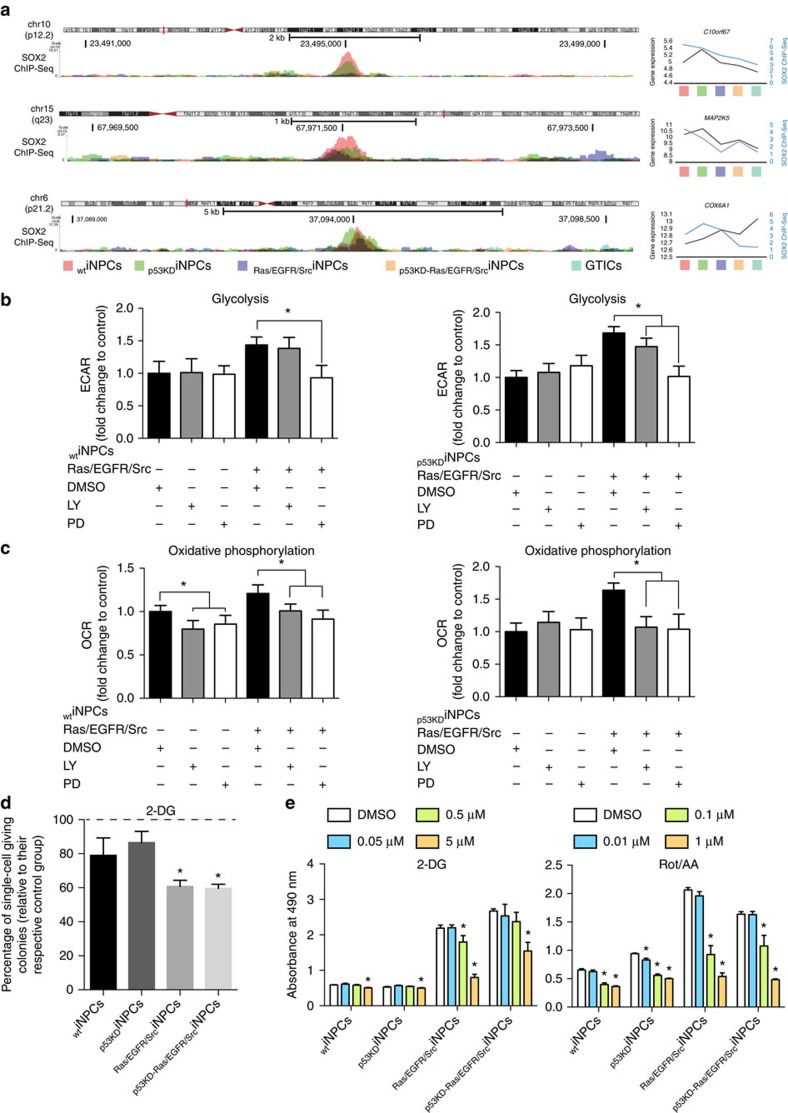
Human iNPCs transformation leads to differential SOX2 binding and metabolic reprogramming. (**a**) Chromatin Immunoprecipitation with SOX2 antibodies followed by deep-sequencing demonstrates differential binding of SOX2 in _WT_iNPCs as compared with transformed iNPCs and primary GTICs. (**b**,**c**) Modulation of PI3K and MAPK signalling pathways with LY294002 (LY) and PD098059 (PD), respectively, reverts the metabolic changes observed in transformed iNPCs as indicated by seahorse analysis. MAPK inhibition restores glycolytic activities (**b**), whereas inhibition of both, PI3K and MAPK, reduced oxidative phosphorylation to the levels observed in _WT_iNPCs (**c**) (*n*=4 group/condition with four technical replicates). (**d**) Inhibition of glycolysis by 2-DG compromises self-renewal properties in transformed iNPCs (*n*=4 group with four technical replicates). (**e**) Chemical induced glycolysis inhibition (2-DG) and ROS production (Rot/AA) compromises transformed iNPC viability in MTS assays (*n*=4 group/condition with four technical replicates). Data are represented as mean±s.d. *P* values were calculated by Student's *t*-test. *P<0.05.

To highlight potential differences between normal iNPCs and GTICs, we analysed the binding of SOX2, a transcription factor common to both _WT_iNPCs and GTICs. ChIP-seq experiments demonstrated differential binding of SOX2 in primary GTICs and transformed GTIC-like cells as compared with _WT_iNPCs (Fig. 3a). Correlation analysis between ChIP and mRNA expression data highlighted a total of approximately eight genes commonly regulated by SOX2 that were both differentially bound as well as differentially expressed between primary GTICs/_p53KD-Ras/EGFR/Src_iNPCs as compared with _WT_iNPCs (Supplementary Data 3, highlighted in red). In agreement with a more undifferentiated phenotype, SOX2 preferentially bound to gene promoters associated to cell dedifferentiation and neural development processes in _p53KD-Ras/EGFR/Src_iNPCs. Interestingly, one gene, *COX6A1*, a gene blocking Bax-induced apoptosis, showed decreased SOX2 binding associated with increased expression in the GTICs and _p53KD-Ras/EGFR/Src_iNPCs compared with the _WT_iNPCs and _p53KD_iNPCs (Fig. 3a). Genes for which decreased SOX2 binding was associated with decreased expression in GTICs and _p53KD-Ras/EGFR/Src_iNPCs compared with _WT_iNPCs and _p53KD_iNPCs were *MAP2K5/ERK5*, whose activities have been associated with tumour development and the acquisition of mesenchymal stem-like properties in cancer cells^34^, and *C10orf67*, a susceptibility locus associated with sarcoidosis and Crohn's disease (Fig. 3a).

### Chemical screening highlights compounds targeting GTICs

**Figure 4:**
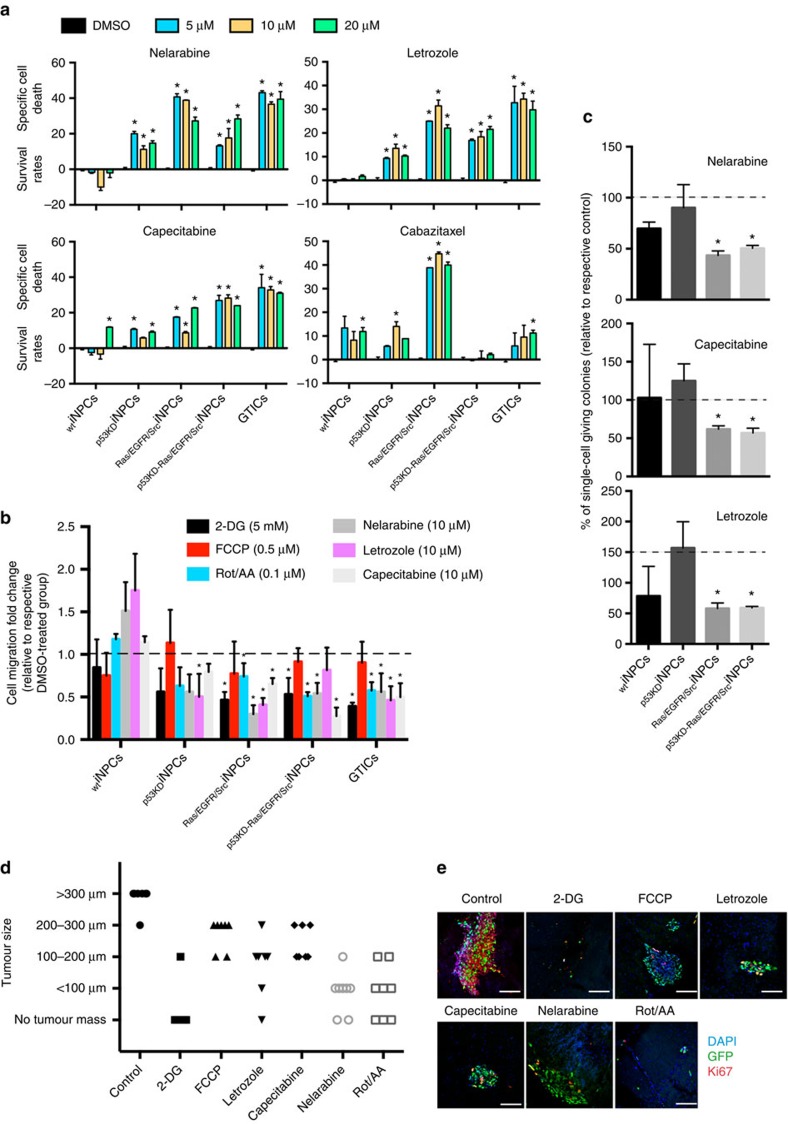
Anti-cancer compound screening identifies chemicals specifically targeting transformed iNPCs and primary GTICs. (**a**) Annexin V staining, as measured by flow cytometry, validates the role of lead compounds at the indicated concentrations and demonstrates a specific effect against primary GTICs and transformed iNPCs for three of the compounds analysed (Nelarabine, Letrozole and Capecitabine). In contrast, Cabazitaxel demonstrated group-dependent effects and targeting of _WT_iNPCs. Data are presented as a ratio to the values obtained in the respective DMSO control groups. Negative values indicate increase viability in where basal Annexin V staining was higher in DMSO samples as compared with those treated with specific compounds; (*n*=3 group/condition with three technical replicates). (**b**,**c**) Treatment of transformed iNPCs and primary GTICs with the identified compounds and metabolic modulators compromises glioma stem cell properties including migration (**b**, *n*=3 group/condition with three technical replicates) and self-renewal potential in single-cell assays (**c**; *n*=3 group/condition with 24 technical replicates). (**d**) Tumour size measured on brain organotypic cultures for each indicated condition. Data are independently plotted for each organotypic brain slice analysed (*n*=>3 condition with technical triplicates). (**e**) Representative pictures of organotypic brain slices injected with primary GTICs and treated with each of the indicated compound. Brain slices were immunostained with the indicated markers as follows: DAPI (blue), GFP (green) and Ki67 (red). Data are represented as mean±s.d. *P* values were calculated by Student's *t*-test or Mann–Whitney test when appropriate. *P<0.05. Scale bars, 100 μm (**e**).

We next tested the suitability of the generated platforms for the testing of chemicals with potential anti-cancer activities. First we investigated the GTIC dependence for hyperactive PI3K/MAPK signalling and their causative role in the acquisition of stem cell properties and metabolic reprogramming. Notably, only MAPK inhibition with PD098059 restored normal glycolytic activities, whereas inhibition of both, PI3K (LY294002) and MAPK signalling, reduced oxidative phosphorylation to the levels observed in _WT_iNPCs (Fig. 3b,c). In addition, chemical inhibition of PI3K and MAPK compromised stem cell self-renewal properties (Supplementary Fig. 7). Next, we focused on metabolic modulation by using: (i) Carbonyl cyanide 4-(trifluoromethoxy)phenylhydrazone (FCCP), a compound dissipating mitochondrial membrane potential; (ii) rotenone in conjunction with antimycin A (Rot/AA), both compounds targeting complexes I and III and leading to the massive production of ROS; and (iii) 2-DG, an analogue glucose acting as a suicide inhibitor. Among all metabolic modulators, inhibition of glycolysis by 2-DG, a compound previously reported for the targeting of gliomas and currently in clinical testing, significantly compromised the self-renewal properties of transformed iNPCs (Fig. 3d). Then, we performed MTS assays to assess cell viability in the presence of metabolic chemical modulators. Interestingly, FCCP did not show any consistent effect across the different groups whereas the use of Rot/AA affected cell viability in all iNPCs (Fig. 3e and Supplementary Fig. 8a). Importantly, inhibition of glycolysis demonstrated a dramatic effect across the different groups of transformed iNPCs, whereas _WT_iNPCs remained largely unaffected (Fig. 3e). In view of these results, we next decided to perform a proof-of-concept screening with 101 FDA-approved anti-cancer compounds (Supplementary Fig. 8b and Supplementary Table 4). MTS assays highlighted 16 different compounds compromising cell viability (Supplementary Fig. 8c and Supplementary Table 4, highlighted in red). Among the different compounds, nelarabine, letrozole and capecitabine, currently under testing for the treatment of gliomas, demonstrated specificity against transformed iNPCs and primary GTICs, whereas cabazitaxel also hampered _WT_iNPC viability (Fig. 4a).

Glioma cell infiltration remains a major cause of tumour relapse and we have previously demonstrated that SFK signalling mediates basal glioma invasion by activating MMP9 (ref. 25). Two-chamber migration assays demonstrated enhanced migratory properties in transformed iNPCs (Supplementary Fig. 9a,b). RNA profiling demonstrated the significant upregulation of *MMP9* in _Ras/EGFR/Src_iNPCs and _p53KD-Ras/EGFR/Src_iNPCs (Supplementary Fig. 9c) and MMP9 inhibition significantly hampered migration (Supplementary Fig. 9b). In addition, all identified anti-cancer compounds and two metabolic inhibitors (2-DG and Rot/AA) hampered the migration of primary GTICs and transformed iNPCs (Fig. 4b). Differentiation of cancer stem cells is thought to give rise to less malignant populations that are presumably more sensitive to chemotherapy, whereas the self-renewing stem cell compartment is thought to contribute to resistance and recurrence^2^,^3^,^8^,^9^,^35^,^36^,^37^. Self-renewal assays indicated that _Ras/EGFR/Src_iNPCs and _p53KD-Ras/EGFR/Src_iNPCs possessed the highest self-renewal potential, whereas _p53KD_iNPCs were more similar to _WT_iNPCs (Supplementary Fig. 9d,e). Treatment with the identified compounds significantly compromised the self-renewal properties of _Ras/EGFR/Src_iNPCs and _p53KD-Ras/EGFR/Src_iNPCs (Fig. 4c). In addition, iNPC-differentiated neural cells demonstrated higher susceptibility to chemical treatments (Supplementary Fig. 10a). Next, we investigated the effect of the identified compounds in established glioma lines. Treatment of U-87 MG and LN229 glioma cells with the different compounds demonstrated the efficient induction of cell death with nelarabine and letrozole, whereas capecitabine was largely ineffective (Supplementary Fig. 10b). Last, we decided to test the effect of the identified compounds alongside metabolic modulators in a more physiological setting. To this end, we relied on the use of *ex vivo* organotypic brain slices^5^,^6^,^38^,^39^. Injection of primary GTICs into 300 μm brain slices resulted in the formation of well-defined tumour masses in less than 8 days (Fig. 4d). Treatment of brain slices indicated a specific effect against primary GTIC tumours with 2-DG, nelarabine and letrozole, demonstrating the highest anti-tumour potential (Fig. 4d,e).

## Discussion

Cancer arises as a consequence of transforming ‘driver' mutations able to initiate tumour formation. Upon tumour initiation, positive selection and clonal progression further leads to the accumulation of ‘passenger' mutations conferring additional growth advantages. Identification of driver mutations might therefore allow not only for the establishment of targeted therapies but also, most importantly, for the elucidation and targeting of the early events underlying cancer formation. Although enormous progress has been made, current bioinformatics approaches tend to focus on diver mutations found in high frequencies while underestimating the role that low frequency mutations might play during carcinogenesis^40^,^41^,^42^,^43^,^44^,^45^.

Since their discovery, iPSCs have been widely used for the modelling of genetic disorders and multiple reports have highlighted the suitability of reprogramming as a platform for drug discovery studies^18^,^36^. In an analogous manner, different strategies have been used for the modelling of human cancer^2^,^3^,^10^,^11^,^12^,^13^,^14^,^15^ including the generation of induced pluripotent cancer cells by reprogramming of cancer cells to a pluripotent state^46^,^44^ and the transformation of differentiated fibroblast by oncogenes and tumour suppressor genes to a cancer stem cell-like phenotype^37^. More recently, conversion of differentiated cancer cells to a multipotent cancer stem cell phenotype upon overexpression of factors defining GTIC identity has been reported^3^. Reprogramming of differentiated glioma cells to glioma stem cells opens the unprecedented opportunity for investigating the dynamic dedifferentiation processes leading to the appearance of GTICs (ref. 3). However, the need for primary tumour material (or glioma cell lines) in where transforming mutations are already present could prevent studies on the role that specific mutations might play during gliomagenesis. Directly addressing the lack of model for studying driver mutations, the Tabar laboratory first reported on the use of NPCs differentiated from human ESCs for studying the role that histone 3.3 mutations play in the formation of pediatric gliomas^2^. Along a similar line, our own results demonstrate the suitability of hiPSCs technologies for the establishment of GTIC-like models recapitulating features observed in adult human gliomas.

In summary, here we report on the application of iPSC technologies for the establishment of tractable human GTIC-like models *in vitro* and *in vivo*. Similar to conventional disease modelling strategies based on the use of hiPSCs, the establishment of hiPSC cancer models might facilitate the future development of novel therapeutics^46^,^47^,^48^.

## Methods

### Reagents and antibodies

The following antibodies were used at the specified concentrations for flow cytometry analysis: mouse anti-human TRA-1–60-FITC 1:10 (560380, BD), mouse anti-human TRA-1–81-APC 1:10 (560793, BD), mouse anti-human CD184 (CXCR4)-APC 1:10 (555976, BD), mouse anti-human CD15-PE and CD15-FITC (561715 and 555401, BD), mouse anti-human CD24-PE 1:10 (560991, BD), mouse anti-human SSEA4-AlexaFluor647 1:10 (560219, BD), mouse anti-human CD44-PE 1:10 (550989, BD), mouse anti-human CD133/2 (293C3)-PE 1:10 (130-090-853, Miltenyi), mouse APC isotype control 1:10 (555751, BD), mouse FITC isotype control 1:10 (555748, BD), mouse PE isotype control 1:10 (555749, BD).

The following antibodies were used at the specified concentrations for immunostainings: sex-determining region Y-box 2 (SOX2; AB5603, 1:500; Chemicon); Oct-3/4 (sc-5279, 1:500; Santa Cruz Biotechnology); NANOG (EB06860, 1:500; Everest Biotech); NANOG (21624, 1:400, abcam); anti-phospho-Akt Ser473 (#9271, 1:50; Cell Signaling); anti-phospho-ERK1/2 Thr202/Tyr204 (#9101, 1:50; Cell Signaling); neuron-specific class III β-tubulin (#MRB-435p and MMS-435p, TUJ-1; 1:500; Covance); microtubule-associated protein 2 (#AB5622, MAP2; 1:500; Millipore); PAX6 (PRB-278P; 1:1000; Covance), glial fibrillary acidic protein (#Z0334, GFAP; 1:500; Dako); α1-fetoprotein (A0008, AFP; 1:400; Dako); calponin (M3556, 1:500; Dako); Ki67 (ab16667; 1:500; abcam); NESTIN (MAB5326, 1:500; Millipore); human nuclear antigen (#MAB1281, HUNU; 1:250, Millipore); O4 (MAB345, 1:200; Milipore), PECAM-1 (#sc-1506, CD31; 1:50; Santa Cruz Biotechnology); 4,6-diamidino-2-phenylindole (DAPI) (5 mg ml^−1^) 1:2,000 (D1306, Invitrogen, Carlsbad, CA); and Alexa Fluor 488 goat anti-mouse (1:1,000, Invitrogen), Alexa Fluor 488 donkey anti-goat (1:1,000, Invitrogen), Alexa Fluor 488 donkey anti-rabbit (1:1,000, Invitrogen) Alexa Fluor 568 donkey anti-mouse (1:1,000, Invitrogen), Alexa Fluor 568 donkey anti-rabbit (1:1000, Invitrogen) and Alexa Fluor 647 chicken anti-rabbit (1:1,000, Invitrogen).

The following chemicals were used at the specified concentrations: TGFβ inhibitor SB431542 10 μM (Reagents Direct; #21-A94); PI3K inhibitor LY294002 10 μM (Sigma-Aldrich, St Louise, MO; #L9908); PD032591 MAPK inhibitor 1 μM (Axon medichem; #Axon140); CHIR99021 3 μM (Axon Medichem; #Axon1386); MMP-9 Inhibitor I 10 nM (Santa Cruz Biotechnology; #sc-311–437); FCCP 0.5 μM, Rotenone+Antimycin A 1 μM and 0.1 μM (Rot/AA) and 2-Deoxy-D-glucose 5 mM (2-DG) (Sigma). DMSO was used as a solvent and as a vehicle control in the respective experiments.

### Cell culture

Neonatal human fibroblasts (HFF-1; ATCC), 293T and the human glioma lines U-87 MG and LN229 (ATCC) were cultured in DMEM containing 10% FBS, 2 mM GlutaMAX (Invitrogen), 50 U ml^−1^ penicillin and 50 mg ml^−1^ streptomycin (Invitrogen). Human foreskin fibroblasts (HFF) were grown in collagen I coated plates (BD biosciences). Human iPSCs were cultured in chemically defined hES/hiPSCs growth media, mTeSR on growth factor reduced matrigel (BD biosciences) coated plates. Briefly, 70–80% confluent iPSCs cells were treated with dispase (Invitrogen) for 7 min at 37 °C and the colonies were dispersed to small clusters and lifted carefully using a 5-ml glass pipette at a ratio of ∼1:4. GTICs, previously reported and characterized^5^, were obtained from BioRep S.r.l. (NS27Z +B AB0789) and maintained in culture as described elsewhere^5^. All cell lines were maintained in an incubator (37 °C, 5% CO2) with media changes every day (iPSCs) or every second day (fibroblasts, 293T cells, GTICs).

### Plasmids

pMX-*OCT4*, pMX-*SOX2*, pMX-*KLF4* and pMX-*cMYC*, were obtained from Addgene (plasmids 17217, 17218, 17219 and 17220, respectively). The shRNA *p53* construct was previously described and validated^49^. Constructs for the expression of mutant oncogenes were obtained from Addgene (RasV12 Neo (w108-1) #22259; EGFR D770-N771 insNPG #11016; EGFR (del3) #11015; src Y527F #13660). Coding sequence for the mutant oncogenes were removed by restriction enzymes and subcloned into a lentiviral backbone with the In-Fusion system according to manufacturer's instructions (Takara/Clontech).

### Retroviral and lentiviral production

Moloney-based retroviral vectors (pMXs) were co-transfected with packaging plasmids (pCMV-gag-pol-PA and pCMV-VSVg) in 293T cells using Lipofectamine 2000 (Invitrogen) according to manufacturer's instructions. Retroviral supernatants were collected 48 h after transfection, passed through a 0.45 μM filter to remove cellular debris and stored at 4 °C for up to 7 days. Lentiviral vectors were co-transfected with packaging plasmids (pMDL, Rev and VSVg) in 293T cells using Lipofectamine 2000 (Invitrogen) according to manufacturer's instructions. Lentiviral supernatants were collected 48 h after transfection, concentrated by ultracentrifugation at 19400, r.p.m. for 2 h, resuspended in PBS and stored at −80 °C.

### Induced pluripotent stem cell generation and subculture

For the generation of human iPS cells, primary HFF were infected with an equal ratio of retroviruses (*Oct4*, *SOX2*, *KLF4* and *c-MYC*) by spinfection of the cells at 1850, r.p.m. for 1 h at room temperature in the presence of polybrene (4 μg ml^−1^). After two serial infections, cells were passaged onto fresh mouse embryonic fibroblasts and switched to hES medium containing DMEM/F12 (Invitrogen) supplemented with 20% Knockout Serum Replacement (Invitrogen), 1 mM L-glutamine, 0.1 mM non-essential amino acids, 55 M -mercaptoethanol and 10 ng ml^−1^ bFGF (preprotech). For the derivation of hiPS cells lines, iPS-like colonies were manually picked and maintained on fresh mouse embryonic fibroblast feeder layers for five passages before being transferred onto Matrigel/mTesR1 conditions (*n*=2 _WT_iPSC clones). iPSCs with a knockdown of p53 were generated as described^49^. Briefly, HFF were infected with lentiviral particles expressing a shRNA against p53 before iPSC reprogramming (*n*=3 _p53KD_iPSC clones).

### Embryoid body differentiation

Embryoid bodies (EBs) were produced from adherent colonies that were enzymatically detached using dispase (0.5 mg ml^−1^) for 15–45 min, collected and then maintained in suspension using ultra low attachment plates (corning) in the presence of EB medium containing DMEM/F12 supplemented with 15% fetal bovine serum, 2 mM L-glutamine, 0.1 mM non-essential amino acids, and 1% penicillin/streptomycin. After 3–4 days, EBs were transferred to 0.1% gelatin-coated polystyrene chamber slides and cultured in differentiation medium (DMEM supplemented with 20% fetal bovine serum, 2 mM L-glutamine, 0.1 mM 2-mercaptoethanol, 0.1 mM non-essential amino acids and 1% penicillin/streptomycin) for 2–3 weeks to allow spontaneous endoderm formation. The medium was changed every other day. For mesoderm differentiation, EBs were maintained on gelatin-coated plate in differentiation medium supplemented with 100 μM ascorbic acid (Sigma). For ectoderm differentiation, EBs were cultured on Matrigel-coated plates in 1% N2 and 0.5% B27 (Invitrogen) medium supplemented with 1 μM retinoic acid for 2–3 weeks.

### Derivation of iNPCs from iPSCs

Neural progenitor cell (NPC) induction was based on a previous report with slight modifications^50^. The day before the start of induction, hiPSCs (_WT_iPSCs and _p53KD_iPSCs) were passaged onto matrigel-coated plates at about 20% confluence and maintained in mTesR overnight. At day 0, culture medium was then switched to Neural Induction Medium 1 (NIM-1: 50% Advanced DMEM/F12 (Invitrogen), 50% Neurobasal (Invitrogen), 1 × N2 (Invitrogen), 1 × B27 (Invitrogen), 2 mM GlutaMAX (Invitrogen) and 10 ng ml^−1^ hLIF (Millipore), 4 μM CHIR99021 (Cellagentech, premade in 10 mM DMSO solution), 3 μM SB431542 (Cellagentech, premade in 10 mM DMSO solution), 2 μM Dorsomorphin (Sigma) and 0.1 μM Compound E (EMD Chemicals Inc.). Cells were treated with NIM-1 for 2 days, and then switched to Neural Induction Medium 2 (NIM-2: 50% Advanced DMEM/F12, 50% Neurobasal, 1 × N2, 1 × B27, 2 mM GlutaMAX, 10 ng ml^−1^ hLIF, 4 μM CHIR99021, 3 μM SB431542 and 0.1 μM Compound E) for another 5 days. The cultures were then split onto Matrigel-coated plates with Accumax (Innovative Cell Technologies) and cultured in Neural Stem cell Maintenance Medium (NSMM) containing 50% Advanced DMEM/F12, 50% Neurobasal, 1 × N2, 1 × B27, 2 mM GlutaMAX, 10 ng ml^−1^ hLIF, 3 μM CHIR99021 and 2 μM SB431542.

### iNPC culture and multilineage neural differentiation

iNPCs were maintained on Matrigel in NSMM and passaged once 80–100% confluent using Accumax. Medium was changed every day. For the initial six passages, iNPCs were treated with 10 μM Y-27632 (Biomol Inc.) during splitting. For neuronal differentiation, ∼1 × 10^5^ human iNPCs cells were plated onto poly-ornithine (Sigma) and laminin coated 35-mm dishes in NSMM medium. After 2 days, medium was switched to Neurobasal media (Invitrogen) supplemented with N2 (Invitrogen), B27 (Invitrogen) and FGF2 (10 ng ml^−1^; Peprotech). Four days later, FGF2 was withdrawn from the medium, and after another 4 days, medium was switched to Neurobasal media supplemented with B27 and brain derived neurotrophic factor (BDNF, 20 ng ml^−1^, R&D Systems). Differentiated cells were maintained up to 4 weeks after FGF2 withdrawal. For astrocyte generation, cells were treated with 5% serum for 2–3 weeks.

### iNPC transformation

Three days before lentiviral transduction, iPSC-derived NPCs (_WT_iNPCs and _P53KD_iNPCs, passages 6–8) were passaged. On the day of transduction, each of the four lentiviral preparations (RasV12, EGFR D770-N771; EGFR (del3) and src Y527F) was thawed on a bed of ice, mixed at a 1:1:1:1 ratio (MOI of 0.75 per virus) in NSMM medium in the presence of polybrene (4 μg ml^−1^). NPCs culture medium was replaced with the lentiviruses containing medium and cells were incubated overnight at 37 °C. The next day, the medium was replaced with fresh NSMM. Transduced iNPCs (_Ras/EGFR/Src_iNPCs, _P53KD-Ras/EGFR/Src_iNPCs were passaged for at least two more passages for stabilization before performing analyses.

### Flow cytometry analysis

For flow cytometry analysis, cells were harvested using TrypLE (Invitrogen), washed once with PBS and further incubated with the corresponding antibodies in the presence of FACS blocking buffer (1 × PBS/10%FCS) for 1 h on ice in the absence of light. After incubation, cells were washed thrice with 1 ml of FACS blocking buffer and resuspended in a total volume of 200 μl before analysis using an LSRII instrument (Becton-Dickinson, Fullertone, CA). A minimum of 10,000 cells in the living population was analysed. Percentages are presented after subtracting isotype background and referring to the total living population analysed. Results are representative of at least two independent experiments with a minimum of two technical replicates per experiment (*n*⩾4) and per condition.

For cell death assays, iNPCs and primary GTICs were treated with 5, 10 and 20 μM for the indicated chemotherapeutic compounds in duplicates in six-well-plates. Human glioma lines (U-87 MG and LN229) were treated with 1 mM for the indicated compounds. 24 h after treatment, cells were dissociated by Accutase (Innovative Cell Technologies, cat. no. AT-104) and Annexin V/PI staining conducted following the manufacturer's recommendations (88-8007-72 Annexin V APC Ebiosciences). PI staining served to exclude dead cells and only Annexin V cells present in the PI negative living cell population (indicative of early apoptosis) were considered for analysis. All stainings were done in biological triplicate with technical triplicates (*n*=9 total) per line and per condition.

### Cell sorting

Transformed iNPCs were sorted for CD15, CD133 and CXCR4 cells using anti-CD15 (130-046-601; Miltenyi biotec), anti-CD133 (130-097-049; Miltenyi biotec) and anti-CXCR4 (130-100-070; Miltenyi biotec) conjugated magnetic beads according to the manufacturer's instructions with slight modifications. Briefly, up to 10^9^ cells were incubated with constant mixing at 4 °C with 100 μl of the corresponding magnetic beads in the presence of 100 μl of Fc-blocking solution in a total volume of 500 μl FACS blocking buffer. After 1 h, cells were sorted by two consecutive rounds of column separation in order to increase purity by applying MACS separation magnets. Shortly, cells were passed through the first MS separation column allowing binding of labelled cells. Non-labelled cells were washed thoroughly with 3 ml fluorescence-activated cell sorting blocking buffer before elution of the labelled fraction. Eluted labelled cells were then subjected to a second purification step as described above. Both positive and negative fractions were collected for further analyses.

### Boyden chamber migration assays

Cells were treated with 10 μg ml^−1^ Mitomycin C (Sigma) for 1 h before the assay. Cells were rinsed once with 1 × PBS (Invitrogen), and treated with TrypLE (Invitrogen) for 5 min at 37 °C. TrypLE was neutralized with DMEM/F12 (Invitrogen)+10% FBS (HyClone). Cells were centrifuged at 1000  r.p.m. for 5 min, and resuspended in their respective basal media. A total of 20,000 cells was seeded onto the upper chamber of each transwell (BD). 1 ml culture media was applied to the lower chamber of each transwell. Migration assay was performed for 24 h in an incubator set at 37 °C in the presence or absence of MMP-9 Inhibitor I (10 nM), the metabolic modulators 2-DG (5 mM), FCCP (0.5 μM), Rot/AA (0.1 μM) and the chemotherapeutic compounds Nelarabine (10 μM), letrozole (10 μM) and capecitabine (10 μM). The cells attached onto the upper side of the transwell filter were removed by a cotton stick. The migrated cells attached onto the bottom side of the transwell filter were fixed with 4% PFA (Sigma), and visualized with 0.5% crystal violet staining (Sigma). Five different areas of each chamber were randomly selected for photography with × 5 objective (Olympus). The average cell number/area was calculated for each chamber. Biological Triplicates with technical triplicates (*n*=9) were performed for each cell line.

### *In vitro* self-renewal and single-cell assays

Transformation assays were conducted by modifying standard soft-agar protocols for the culturing of iNPCs similar to what was previously described^51^. Briefly, 2 × 10^4^ cells were individualized, embedded into 2% matrigel and seeded in 60-mm dishes. Biological triplicates with technical triplicates (*n*=6) were performed for each tested cell line and condition.

For single-cell self-renewal assays the experimental setup was modified as follows: undifferentiated (sorted or unsorted) iNPCs were dissociated and plated into matrigel-coated 96-well plates as single cells as described^52^. Each condition was done in 24 technical replicates with a minimum of three biological duplicates (*n*⩾62). One day after plating, all wells were treated with the respective compounds as indicated. After 21 days, the colony number was evaluated by microscopy analysis using an upright microscope (Olympus). For single-cell self-renewal assays in the presence of PI3K (LY294002; 5 μM) and MAPK (PD98059; 5 μM) inhibitors, individual cells were plated as described above and the media supplemented with the respective chemicals or the solvent control (DMSO) at the indicated concentration and colonies counted after 21 days. Biological triplicates with technical triplicates (*n*=9) were performed for each tested cell line and condition.

Single-cell kill assays were conducted by plating individual iNPCs onto 96-well plates as single cells before differentiation. Differentiation into neuronal lineages was performed as described above in the presence of the indicated chemicals or the respective DMSO controls. Cell survival was estimated with trypan blue after differentiation over 28 days. Each condition was done in 24 replicates with a minimum of two independent times (*n*⩾48).

### Immunoblotting

Cells were solubilized in lysis buffer (50 mM Tris-HCl (pH 7.4), 10% Glycerol, 1% (v/v) Triton X-100, 100 mM NaCl, 0.5 mM MgCl_2_, 1 mM Na_3_VO_4_, 10 μg ml^−1^ aprotinin, 1 mM PMSF, 1 mM pepstatin and leupeptin), and the resulting lysates were prepared by centrifugation at 16,000*g* for 15 min at 4 °C. The protein concentration of cell lysates was tested using the protein assay reagent (Bio-Rad) before immunoblotting. Protein samples were separated on SDS–polyacrylamide gel electrophoresis, and transferred onto PVDF membrane filters (PALL). The following primary antibodies were used according to the manufacturer's instructions: anti-phospho-Akt Ser473 (#9271, Cell Signaling), anti-Akt (#9272, Cell Signaling), anti-phospho-ERK1/2 Thr202/Tyr204 (#9101, Cell Signaling), anti-ERK1/2 (#9102, Cell Signaling), anti-NANOG (#ab21624, Abcam), anti-α-Tubulin (#T5168, Sigma) and anti-β-Actin (#sc-47778, Santa Cruz). Uncropped images of the most important immunoblots are provided in Supplementary Fig. 11.

### RNA isolation and real-time PCR analysis

Total cellular RNA was isolated using Trizol Reagent (Invitrogen) according to the manufacturer's recommendations. An amount of 1 μg of DNAse1 (Invitrogen) -treated total RNA was used for cDNA synthesis using the iScript cDNA synthesis kit for PCR with reverse transcription (BioRad). Real-time PCR was performed using the SYBR Green Supermix (BioRad). The levels of expression of respective genes were normalized to corresponding *GAPDH* values and are shown as fold change relative to the value of the control sample. All samples were done in biological triplicate with a minimum of two technical replicates (*n*⩾5). The list of the primers used for real-time PCR experiments are listed in the Supplementary Table 5.

### Metabolic flux analysis

Analysis of oxygen consumption rate (OCR) and extracellular acidification rate (ECAR) was performed using a Seahorse extracellular flux XF96 analyser (Seahorse Bioscience, Billerica, MA) in accordance with manufacturer's instructions. Briefly, cells were seeded at a density of 1.5 × 10^4^ cells per well in Matrigel-treated extracellular flux 96-well culture microplates in 150 μl of corresponding cell culture media and incubated overnight at 37 °C. The next day, before analysis, culture media was replaced with 150 μl of unbuffered assay media and cells were incubated for 1 h at 37 °C for pH and temperature stabilization. Analysis of OCR and ECAR was performed simultaneously both at basal conditions and after injections of the inhibitors in the XF Cell Mito Stress Test Kit (1 μg ml^−1^ Oligomycin, 0.5 μM FCCP, 1 μM Antimycin A+1 μM Rotenone (Sigma)). To evaluate the effect of the respective PI3K and MAPK inhibitors LY294002 and PD0325901 on cellular metabolism, cells were incubated overnight (12 h) in the presence of 10 μM LY294002 or 1 μM PD0325901. The next day, before analysis, culture media was replaced and analysis of OCR and ECAR was performed as described above. Each condition was done in four technical replicates with a minimum of four biological replicates (*n*⩾16).

### MTS assays

Analysis of cellular viability in the presence of respiratory inhibitors was performed using MTS assay (Promega, G3580) according to the manufacturer's instructions. Briefly, cells were seeded at a density of 2 × 10^4^ cells per well in Matrigel-treated 96-well culture microplates in 100 μl of culture media and incubated for 4 h at 37 °C. Next, the mitochondrial respiratory inhibitors FCCP or 0.5 μM Antimycin A+0.5 μM Rotenone (Sigma) were added at the indicated concentrations. After 24 h of compound/DMSO treatment, 20 μl of MTS reagent was applied to each well of 96-well plate. Absorbance at 490 nm was recorded after 2 h incubation. Sextuplets were prepared for each condition.

### ROS measurements

Mitochondrial-associated ROS levels and total intracellular ROS levels were measured by staining cells with MitoSOX and H_2_DCFDA. For mitochondrial superoxide, cells were washed with PBS solution, trypsinized and resuspended in PBS in the presence of 5 μM MitoSOX (Invitrogen) for 15 min at 37 °C. Subsequently, cells were washed twice with PBS solution containing 10% FBS and analysed by FACS using an LSRII instrument (Becton-Dickinson). For total intracellular ROS, cells were washed with PBS solution and incubated in HBSS solution in the presence of 5 μM H_2_DCFDA (Invitrogen) for 30 min at 37 °C. Afterwards, the HBSS solution was replaced with regular media and cells were incubated for an additional 1 h at 37 °C. Lastly, cells were washed with PBS solution, trypsinized and resuspended in PBS solution containing 10% FBS for FACS analysis using an LSRII instrument (Becton-Dickinson). As a positive control, cells were incubated in the presence of the mitochondrial inhibitor Antimycin A (Sigma) at a concentration of 0.1 μM for 30 min at 37 °C to induce ROS production. Each condition was done in two technical replicates with three biological replicates (*n*=6).

### Mitochondrial staining

For mitochondrial staining, cells were seeded on Matrigel-treated cover slips at the appropriate densities. The next day, cells were incubated for 30 min at 37 °C in regular media in the presence of 200 nM MitoTracker Red (Invitrogen) for staining of mitochondria and 1 μg ml^−1^ DAPI (Invitrogen) to stain the cell nuclei. Thereafter, cells were fixed in 4% paraformaldehyde and visualized by a confocal microscopy (Zeiss, Oberkochen, Germany) with excitation at 578 nm and emission at 598 nm for MitoTracker Red, and excitation at 359 nm and emission at 461 nm for DAPI detection. Each condition was done in two technical replicates with three biological replicates (*n*=6).

### Gas chromatography/mass spectrometry/stable isotope labelling

For GC–MS profiling analysis of intracellular metabolites, cells were seeded and cultured in 6-well dishes (to about 1 million cells / 0.5 mg cell protein per well). Cells were washed quickly three times with cold PBS, and 0.45 ml cold methanol (50% v/v in water with 20 μM L-norvaline as internal standard) was added to each well. Culture plates were transferred to dry ice for 30 min. After thawing on ice, the methanol extract was transferred to a microcentrifuge tube. Chloroform (0.225 ml) was then added, the tube was vortexed and centrifuged at 10,000*g* for 5 min at 4 °C. The upper layer was transferred to another microcentrifuge tube, dried in a centrifugal evaporator and derivatized with 30 μl *O*-isobutylhydroxylamine hydrochloride (20 mg ml^−1^ in pyridine, TCI) for 20 min at 80 °C, followed by 30 μl *N*-tert-butyldimethylsilyl-*N*-methyltrifluoroacetamide (Sigma) for 60 min at 80 °C. After cooling overnight at 4 °C, the mixture was then transferred to SHIMADZU GCMS-QP2010 Plus for analysis.

GC–MS protocols and analysis were similar to those described before^53^, except a modified temperature gradient was used for GC: Initial temperature was 130 °C, held for 4 min, rising at 6 °C min^−1^ to 243 °C, rising at 60 °C min^−1^ to 280 °C, held for 2 min. Data were processed as before with quantification against varied amounts of standard mixtures run in parallel performed using MetaQuant. Quantities were corrected for recovery using the L-norvaline internal standard.

For labelling experiments, cells were seeded as described above. Then cells were changed to NSMM, with 50% ^13^C-glucose for labelling. The glucose was isotopically labelled at all six carbon positions ([U- ^13^C_6_] glucose). After 24 h of incubation, labelled cells were rinsed with cold PBS. Cells were then processed for GC/MS sample extraction and derivatization, and analysed as described before^53^,^54^.

For normalization, protein quantification was determined. Cells from separate and replicate wells for GC/MS were rinsed once with ice-cold PBS and lysed in ice-cold lysis buffer (25 mM Tris (pH 8.0), 100 mM NaCl, 1% TritonX-100 and 10% Glycerol) and one tablet of EDTA-free protease inhibitors (Roche; per 25 ml). The soluble fractions of cell lysates were isolated by centrifugation at 13,000  r.p.m. for 15 min. Protein concentration was determined by the Bradford method with Bio-Rad DC Protein Assay Kit (Hercules, CA, USA).

### Animals

Murine experiments presented hereafter were conducted with approval of The Salk Institute Institutional Animal Care and Use Committee (IACUC), under the protocol#08–025. NOD.Cg-PrkdcscidIl2rgtm1Wjl /SzJ mice (or NOD-Scid IL2rγnull abbreviated as NSG; age, 7 weeks; males and females; weight, 20 g) were purchased from the Jackson Laboratory, housed in air-flow racks on a restricted access area and maintained on a 12-h light/dark cycle at a constant temperature (22±1 °C). For all experiments involving the use of animals, no inclusion/exclusion criteria were applied in the present study.

### Teratoma assay

Severe combined immune NSG male mice (*n*=2 animal/iPS clone; 5 clones total), ∼8 weeks old, were injected with iPSCs (1 million for each injection site, approximately) subcutaneously in the testicular parenchyma. Additionally, undifferentiated iPSCs were stereotactically injected into the brain of immunocompromised NSG mice (*n*=2 animal/iPS clone; five clones total). Mice were sacrificed 8 weeks after the injections or when a tumour was detected by palpation or when animals displayed signs of distress, whichever came first. Teratoma formation was assessed by immunofluorescence techniques. Randomization was unnecessary and no blinding was done.

### *In vivo* tumour formation

For cell preparation, cells were rinsed once with 1XPBS (Invitrogen), and treated with TrypLE (Invitrogen) for 5 min at 37 °C. TrypLE was neutralized with DMEM/F12 (Invitrogen)+10% FBS. Cells were centrifuged at 1000, r.p.m. for 5 min, and resuspended in PBS (2 × 10^5^ μl^−1^ or 500 μl^−1^ for 5 × 10^5^ and 500 transplanted cells, respectively). Cells were kept on a bed of ice before transplantation. For cell transplantation, anaesthetized NSG mice were inserted in a stereotactic frame, the skull surface was exposed and a hole was drilled at the appropriate site to allow single/unilateral (right hemisphere) injection of the cell preparations. Antero-posterior (AP), lateral (L) and vertical (V) coordinates (in mm) for cell transplantations were taken relative to the bregma: AP+0.5, L+2, V −1.5. For cell injections, a 10 μl Hamilton syringe was used and the flow rate set at ∼0.5 μl min^−1^ (2.5 μl or 0.5 μl animal for 5 × 10^5^ and 500 transplanted cells, respectively). For each condition, five animals were injected. Randomization was unnecessary and no blinding was done.

For secondary transplantation experiments, primary recipient animals were sacrificed by carbon dioxide (CO2) overdose and their brains immediately removed. The tumour was then extracted under stereomicroscope with surgical tools, in sterile conditions. The tumour mass was cut into small pieces and placed in TrypLE solution for 15 min at 37 °C. The tumour was fully dissociated by mechanical dissociation to obtain a single-cell solution, and passed through a 0.45 μm filter. Then, the cells were prepared and injected into immunocompromised mice as described above. Of note, tumour extraction, cell preparation and cell injection were performed the same day. For each condition, five animals were injected. Randomization was unnecessary and no blinding was done.

### Animal perfusion/tissue sectioning

Animals were daily monitored and sacrificed/perfused when showing cues of sickness (that is, dehydrated, lethargic, scruffy and/or hunched). For perfusion, mice were sacrificed by carbon dioxide (CO2) overdose and then transcardially injected with 20 ml ice-cold saline (0.9^0^/_00_ NaCl), followed by 50 ml ice-cold 4% paraformaldehyde (PFA, pH 7.4; 4 °C). Brains were then extracted and post-fixed in PFA overnight at 4 °C. Thirty-six to forty hours before sectioning, brains were cryoprotected in a 30% sucrose solution. Frozen coronal sections (35 μm thickness) were cut with a cryostat (Leica) and were either collected onto positively charged slides or kept at −20 °C into an ethylenglycol-based cryoprotectant solution until being processed for immunostainings. Before performing cryosections, whole brain pictures were taken with a camera.

### Haematoxylin/eosin staining

Tissue sections on positively charged slides were processed for haematoxylin-eosin staining by dipping the slides in a series of baths as follow: distilled water (30 s), haematoxylin (5 min); distilled water (1 min); 0.5% eosin Y (2 min); 95% Ethanol (30 s × 2); 100% ethanol (30 s × 2); and xylene (30 s × 2). Depex mounting medium and glass cover slips were used to mount the slides. Slides were analysed by using an upright microscope (Olympus).

### Immunofluorescence analyses

Briefly, cells were washed thrice with PBS and fixed using 4% PFA in 1 × PBS for 12 min and then washed three times in PBS. For tissue analysis, fixed floating sections maintained in a cryoprotectant solution were washed in PBS before being immunostained. Fixed cells/tissues were blocked and permeabilized for 1 h at RT with 3% BSA/5% appropriate serum/1 × PBS in the presence of 0.1% Triton X-100 and 0.3 M glycine. Subsequently, cells/tissue sections were incubated with the indicated primary antibody either for 1 h at room temperature or overnight at 4 °C. Cells/tissue sections were then washed thrice with 1 × PBS and incubated for 1 h at RT with the respective secondary antibodies and 20 min with DAPI. Cells/tissue sections were washed thrice with 1 × PBS before analysis. Cells/tissue sections were analysed by confocal microscopy. Confocal image acquisition was performed using a Zeiss LSM 780 or LSM 710 laser scanning microscope (Carl Zeiss).

### Gene expression analysis

For gene expression analyses, RNA was purified using the RNeasy Plus mini kit (74134, Qiagen). Purity and integrity of the RNA samples were assessed by spectrophotometry and nanoelectrophoresis using the NanoDrop ND-1000 spectrophotometer (NanoDrop Technologies, Wilmington, DE) and the Nano lab-on-a-chip assay for total eukaryotic RNA using Bioanalyser 2100 (Agilent Technologies, Palo Alto, CA), respectively. Only samples with high purity and high integrity were subsequently used in microarray experiments.

Microarray expression profiles were obtained using the Affymetrix GeneChip Human Gene 1.0 ST Array (Affymetrix, Santa Clara, CA). Amplification, labelling and hybridizations were performed according to protocols from Ambion (Ambion/Applied Biosystems, Foster City, CA, USA) and Affymetrix (Affymetrix Inc.). Briefly, 200 ng of total RNA were amplified using the Ambion WT Expression Kit (Ambion/Applied Biosystems), labelled using the WT Terminal Labeling Kit (Affymetrix) and then hybridized to Human Gene 1.0 ST Array (Affymetrix) for 16 h at 45 °C and 60  r.p.m. in a GeneChip Hybridization Oven 640. Following hybridization, the array was washed and stained in the Affymetrix GeneChip Fluidics Station 450. The stained array was scanned using an Affymetrix GeneChip Scanner 3000 7 G, generating CEL files for each array. Normalized and log transformed gene expression values were filtered using a variance filter (σ/σ max) of 0.2. Each variable was standardized by subtraction of its mean value and division by its standard deviation across all samples. One _p53KD-Ras/EGFR/Src_iNPC outlier sample was removed from further analysis. Differential expression analysis was carried out using ANOVA in Qlucore Omics Explorer 2.3. Probes with a *q* value <0.01 were considered differentially expressed. The differentially expressed probes were then clustered using hierarchical clustering and only the probes (*n*=463) that displayed high correlation between the GTICs and _p53KD-Ras/EGFR/Src_iNPC samples were displayed in the heatmap. Pathways analysis was performed using IPA software (Ingenuity Systems, build v. 308606 M, content v. 18488943).

### Chip-Seq and methylation analysis

ChiP-Seq data was mapped and loaded into Seqmonk tools (Babraham Institute) and duplicate reads were removed. Peaks were defined as a contiguous set of reads that were enriched by at least 10-fold. A 50 base pair gap was allowed to prevent large peaks from being broken into multiple small peaks. The number of reads in each peak were counted, corrected for total read count and log transformed. An intensity difference test was used to identify differential SOX2-binding sites. An intensity difference test is a pairwise statistical test using the general distribution of the data and tests whether a particular point is likely to be an outlier when compared with the overall distribution of the data. The test takes each point and tests a subset of points from the pair of samples being examined and then constructs a distribution of the differences between the two data sets. The distribution is then compared with a normal distribution allowing for a *P* value to be calculated. A FDR of 0.05 was used as a cutoff. Genes within 100 kb of the differentially bound peak were considered to be associated with SOX2-binding events.

For methylation analyses, DNA was purified by using DNeasy kit (69504, Qiagen), quantified (Qubit dsDNA BR Assay, Life Technologies) and bisulfite-converted (EZDNA Methylation Kit, Zymo Research) according to the manufacturer's protocol. Bisulfite-converted DNA was then labelled and hybridized to the Infinium Human Methylation 450 K beadchip (Illumina) and scanned on a HiScan (Illumina). Samples were normalized and filtered using the statistical programming language R (http://www.r-project.org/) (v.3.0.1) and the R package minfi (v.1.6.0). Briefly, samples were first control-normalized using Illumina's internal bead controls and probes with a detection *P* value >0.01 in at least one sample were discarded. The samples were then SWAN normalized using the minfi package and *β* values (Methylated allele intensity/ Unmethylated allele intensity+methylated allele intensity) were exported. Probes with less than a max β—min β of 0.5 were removed.

### Compound screening

101 anti-cancer compounds were provided by the National Cancer Institute-chemotherapeutic agents repository. A full list is provided in Supplementary Table 7. For MTS assays, 96-well plates were coated with matrigel (BD, cat. no. 354230) in a 37 °C cell culture incubator for 2 h. iNPCs and GTICs were enzymatically dissociated by Accutase (Innovative Cell Technologies, cat. no. AT-104). Live cell number was counted using a hemocytometer in the presence of Trypan Blue (Gibco, cat. no. 15250). For each iNPC line, 30,000 live cells were resuspended in 50 μl NSMM and seeded into one well of 96-well plate. For GTICs, 15,000 live cells were resuspended in 50 μl NSMM and seeded into one well of 96-well plate. The cells were allowed to grow in a 37 °C cell culture incubator for 24 h before compound/DMSO treatment. Compound/DMSO were diluted in NSMM (final concentration of 10 μM) and added into each well to make a 100-μl final volume. After 24 h compound/DMSO treatment, 20 μl MTS (Promega, G3580) was applied to each well of 96-well plate. Absorbance at 490 nm was recorded after 2 h incubation. Quadruplets were prepared for one compound/line. For Flow Cytometry analysis, cells were treated in the presence of 5, 10 and 20 μM in duplicates in six-well-plates. 24 h after treatment cells were dissociated by Accutase (Innovative Cell Technologies, cat. no. AT-104) and Annexin V/PI staining conducted following the manufacturer's recommendations (88-8007-72 Annexin V APC Ebiosciences). PI staining served to exclude dead cells and only Annexin V cells present in the PI negative living cell population (indicative of early apoptosis) were considered for analysis. All stainings were done in biological and technical triplicates per line and per condition.

### Brain organ cultures

Brain organotypic slices were obtained from adult male NSG mice as previously described^55^,^56^. Briefly, 3–8, 300 μm slices per murine brain were obtained with a Leica VT1200 microtome and cultured over 0.4 μm air–liquid interface membranes (Millipore) for 24 h. 200,000 cells were then injected and tumour formation allowed for the next 72 h. Brain slices were then incubated in the presence of the indicated chemicals or the respective DMSO control for 72 h before immunofluorescence analysis. Each condition was done in two technical replicates with a minimum of three biological replicates (*n*⩾6).

### Statistical evaluation

Statistical analyses were performed by using SPSS/PC+statistics 11.0 software (SPSS Inc., Chicago, IL). All data presented a normal distribution. Statistical significance was evaluated using standard unpaired Student's *t* test (two-tailed, 95% confidence intervals, *P*<0.05) with Welch's correction when appropriate. For multiple comparison analysis, one-way ANOVA with Dunnett's or Bonferroni correction post-test was applied when appropriate (*P*<0.05). Comparisons of groups with small sample size (*n*<6) were performed as follows: (i) Mann–Whitney test (two-sided, 95% confidence level; *P*<0.05) was used; (ii) Kruskal–Wallis test with Dunn's post-test (*P*<0.05) has been applied for multiple comparisons. All data are presented as mean±s.d. and represent a minimum of two independent experiments with at least two technical replicates.

## Additional information

**Accession codes:** Differential ChiP-Seq, Methylation and Gene Expression data have been deposited in GEO under the accession code GSE67286.

**How to cite this article:** Sancho-Martinez, I. *et al*. Establishment of human iPSC-based models for the study and targeting of glioma initiating cells. *Nat. Commun.* 7:10743 doi: 10.1038/ncomms10743 (2016).

## Supplementary Material

Supplementary Information Supplementary Figures 1-11 and Supplementary Tables 1-5

Supplementary Data 1 mRNA expression levels of genes commonly regulated in transformed human NPCs and primary GSCs

Supplementary Data 2 Ingenuity cluster analysis gene list

Supplementary Data 3 Correlation analysis of ChIPseq and mRNA expression data
