# Supplementary material for: Establishment of human iPSC-based models for the study and targeting of glioma initiating cells
Source: Nat Commun. 2016 Feb 22;7:10743. doi: 10.1038/ncomms10743 (PMC4764898; doi:10.1038/ncomms10743)
Supplement: Supplementary Information — Supplementary Figures 1-11 and Supplementary Tables 1-5 [file ncomms10743-s1.pdf]

Supplementary Figures

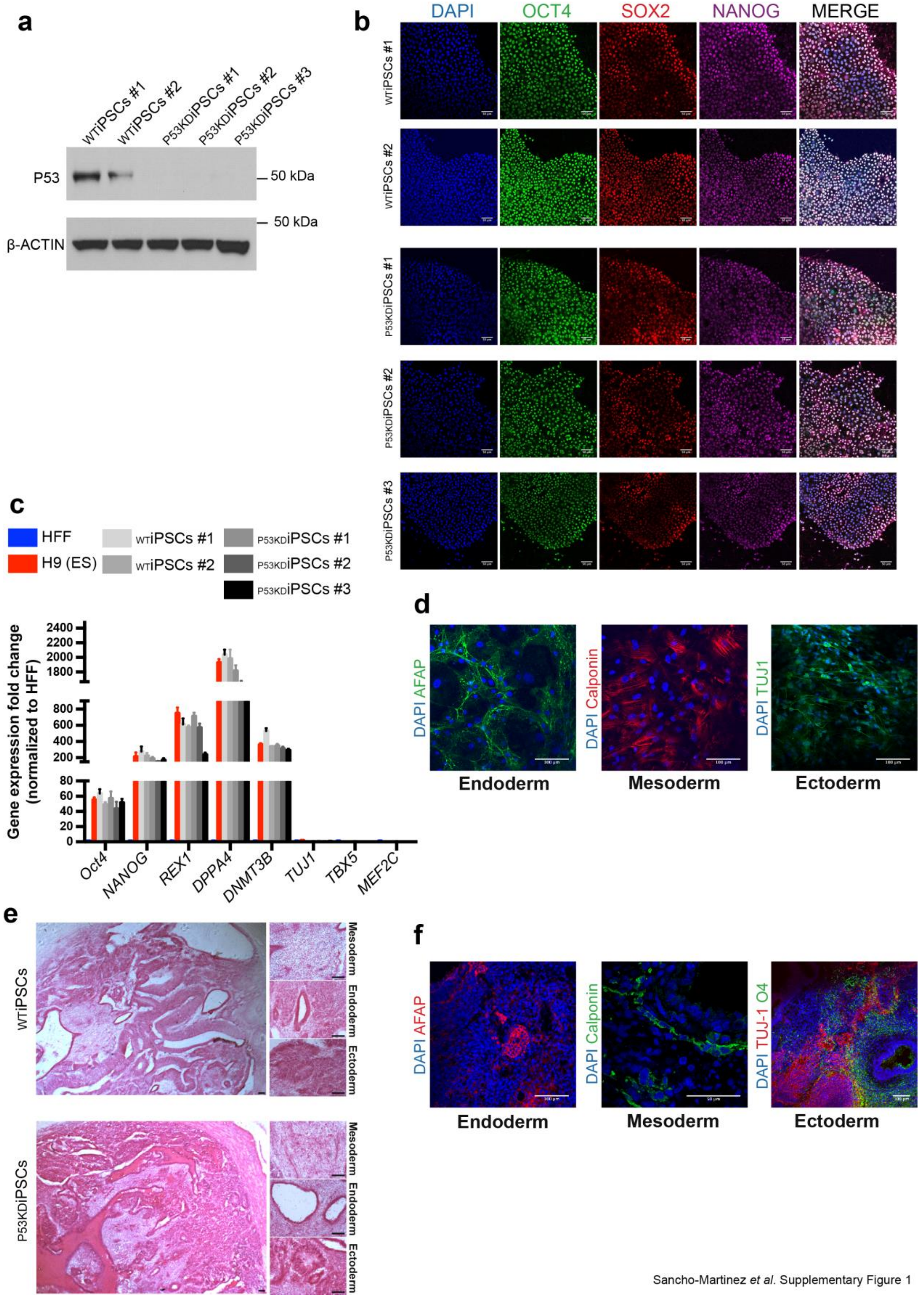

Sancho-Martinez *et al.* Supplementary Figure 1

Supplementary Figure 1. **Characterization of the generated hiPSC lines**

a) Western blot analysis indicating the efficient downregulation of p53 upon knockdown in three hiPSC lines ($_{p53KD}$iPSC#1, #2 and #3). Two « wild-type » hiPSC lines ($_{WT}$iPSC#1 and #2) were used as controls. **b)** Representative pictures of immunofluorescence analysis demonstrating expression of the indicated pluripotency markers in the generated hiPSC lines. **c)** qPCR analysis demonstrating the endogenous upregulation of pluripotency markers and absent expression of markers typical of neurons (TUJ1), mesoderm derivatives (TBX5) and cardiac cells (MEF2C) (n=2/line with technical triplicates). **d)** Representative pictures demonstrating that Embryoid Bodies generated from the hiPSC lines can give rise to cells belonging to all three germ layers upon in vitro directed differentiation (n=2 animal/iPS clone, 5 clones total). Immunofluorescence analysis was performed against the indicated markers. **e)** Hematoxylin-Eosin staining highlighting the presence of well-defined teratomas upon intracerabral injection of undifferentiated $_{WT-}$ and $_{p53KD-}$ hiPSCs into immunocompromised mice. **f)** Representative pictures of immunofluorescence analysis highlighting the presence of differentiated derivatives of the three germ layers in teratomas obtained upon intracerebral injection of $_{WT-}$ and $_{p53KD-}$ hiPSCs. Data are represented as mean +/- SD. Scale bars: 50µm (b); 100µm (d); 200µm (e); 50 and 100 µm as indicated (f).

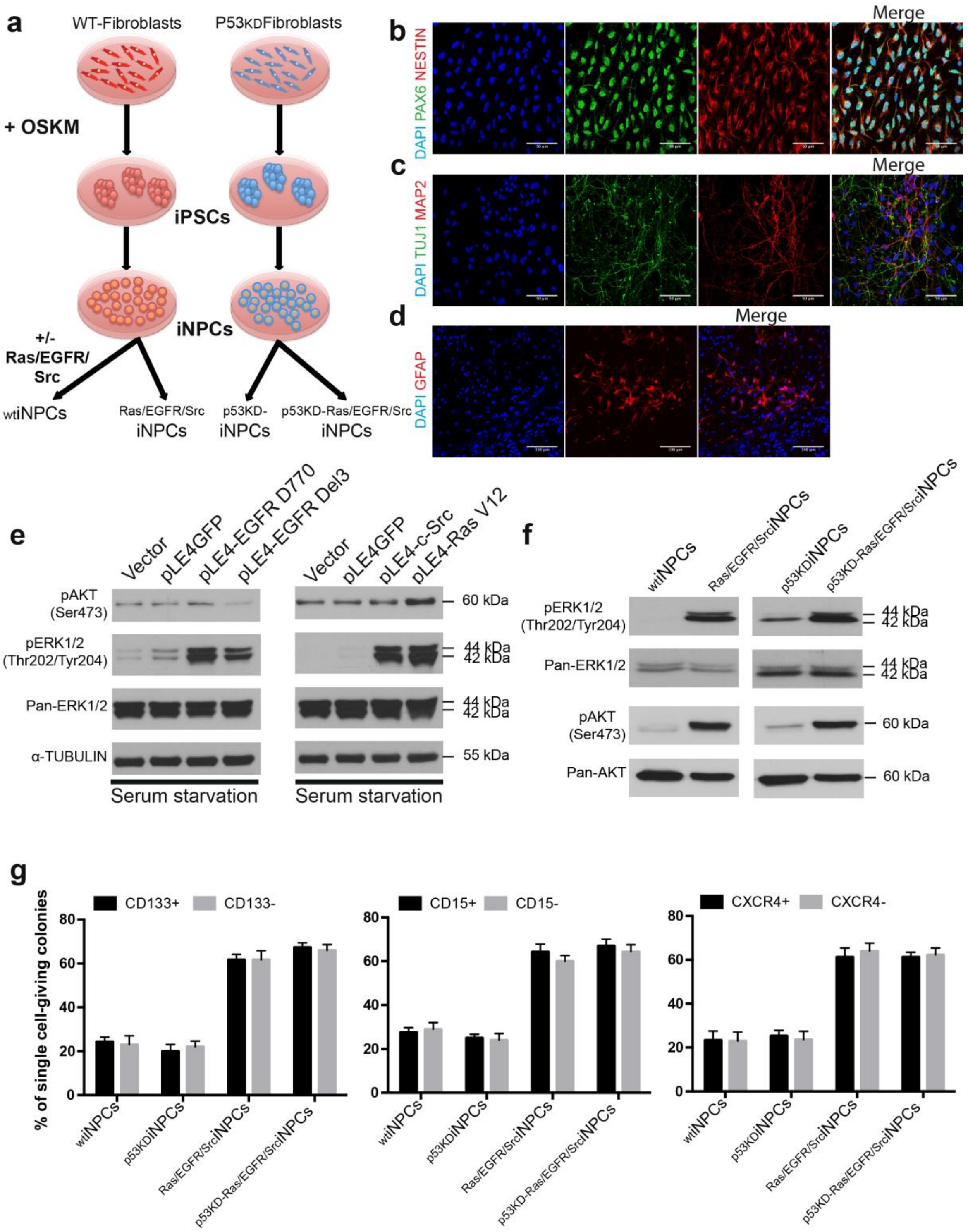

Sancho-Martinez *et al.* Supplementary Figure 2

Supplementary Figure 2. **Transformation of human iNPCs to GTIC-like cells upon disruption of key signaling pathways**

a) Schematic representation depicting the strategies used for the generation of transformed human iNPCs. OSKM stands for *Oct4/SOX2/KLF4/c-MYC*. **b-d)** Representative immunofluorescence pictures demonstrating the neural progenitor identity of hiPSC-derived iNPCs as indicated by PAX6 and NESTIN expression (b). Newly generated iNPCs retained the capacity to differentiate towards the neuronal lineage as indicated by TUJ1 and MAP2 expression (c), as well as towards the astrocyte lineage as indicated by GFAP expression (d). Cell nuclei were counterstained with DAPI. **e)** Western blot analysis demonstrating the effect of each individual construct in 293T cells under serum starvation. **f)** Dysregulation of p53 and/or PI3K and MAPK signaling in iNPCs results in the hyperactivation of AKT and ERK upon transduction with the different constructs. **g)** Transformation of iNPCs results in the acquisition of functional cancer stem cell properties regardless of marker expression. Sorting of different iNPC cell populations based on the indicated markers demonstrated comparable self-renewal potential in single-cell assays (n=3/group with 24 technical replicates). Data are represented as mean +/- SD. *p*-values were calculated by Student's *t*-test. *p<0.05, n=>3. Scale bars: 50µm (b,c) and 100µm (d).

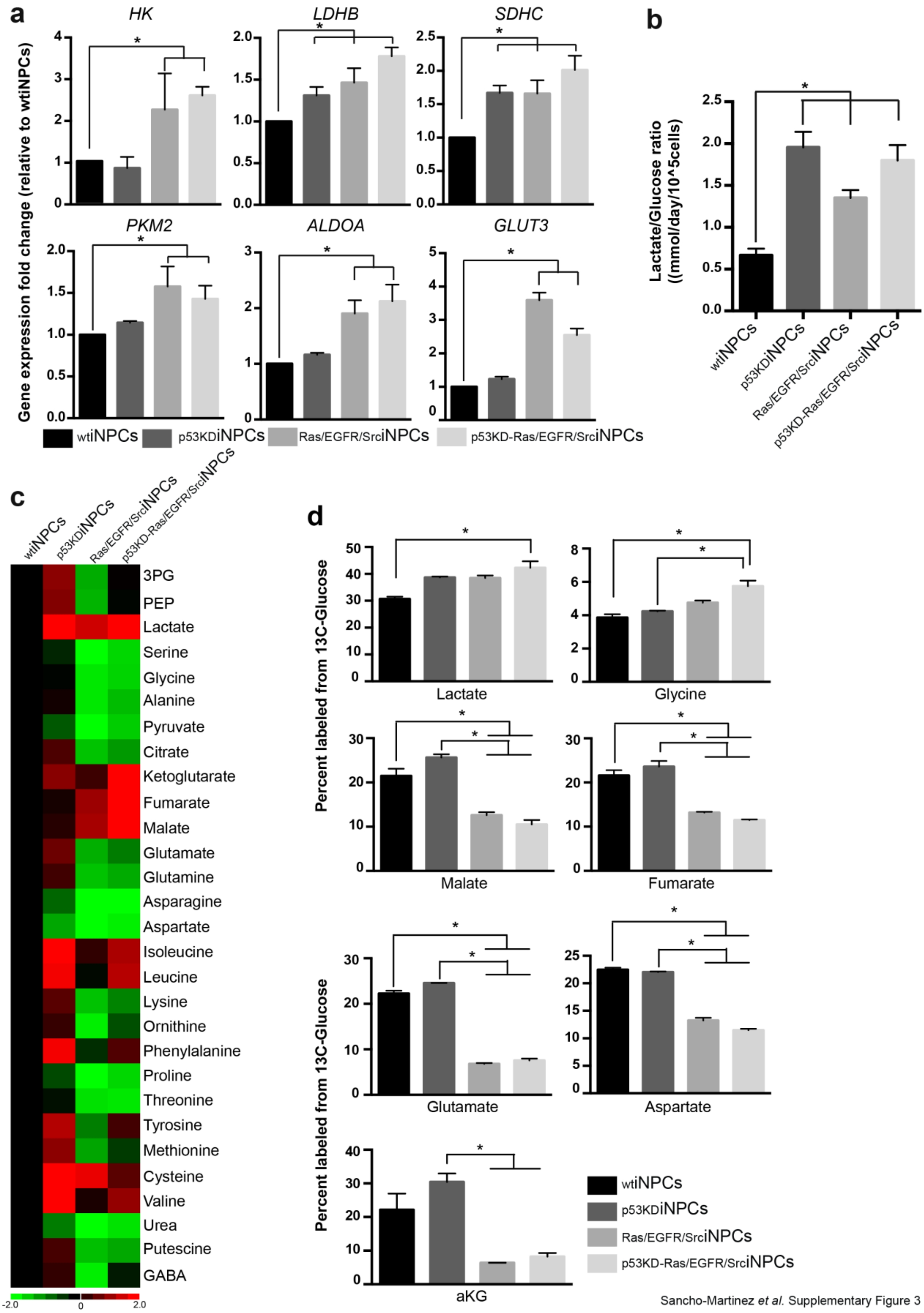

Sancho-Martinez *et al.* Supplementary Figure 3

Supplementary Figure 3. **Transformation of human iNPCs induces metabolic reprogramming**

a) qPCR analysis highlighting the upregulation of metabolic genes, related to GTICs, in transformed iNPCs (n=>3/group with technical triplicates). **b)** Transformed iNPCs present an increase ratio of lactate production indicating the acquisition of a more glycolytic metabolism (n=4/group with 4 technical replicates). **c)** Heat-map highlighting the metabolite levels found between different iNPC groups upon Mass Spectrometry analysis. Please note that $_{p53KD}$iNPCs resemble to a greater state $_{WT}$iNPCs whereas more profound changes are observed in the $_{Ras/EGFR/Src}$iNPCs and $_{P53KD-Ras/EGFR/Src}$iNPCs groups. **d)** U-13C labeling followed by Mass Spectrometry analysis indicated that transformation of human iNPCs compromised carbon input into the TCA cycle and Glutamine production (n=3/group with technical duplicates). Data are represented as mean +/- SD. *p*-values were calculated by Student's *t*-test or Mann-Whitney test when appropriate and represented as follows: *$p<0.05$.

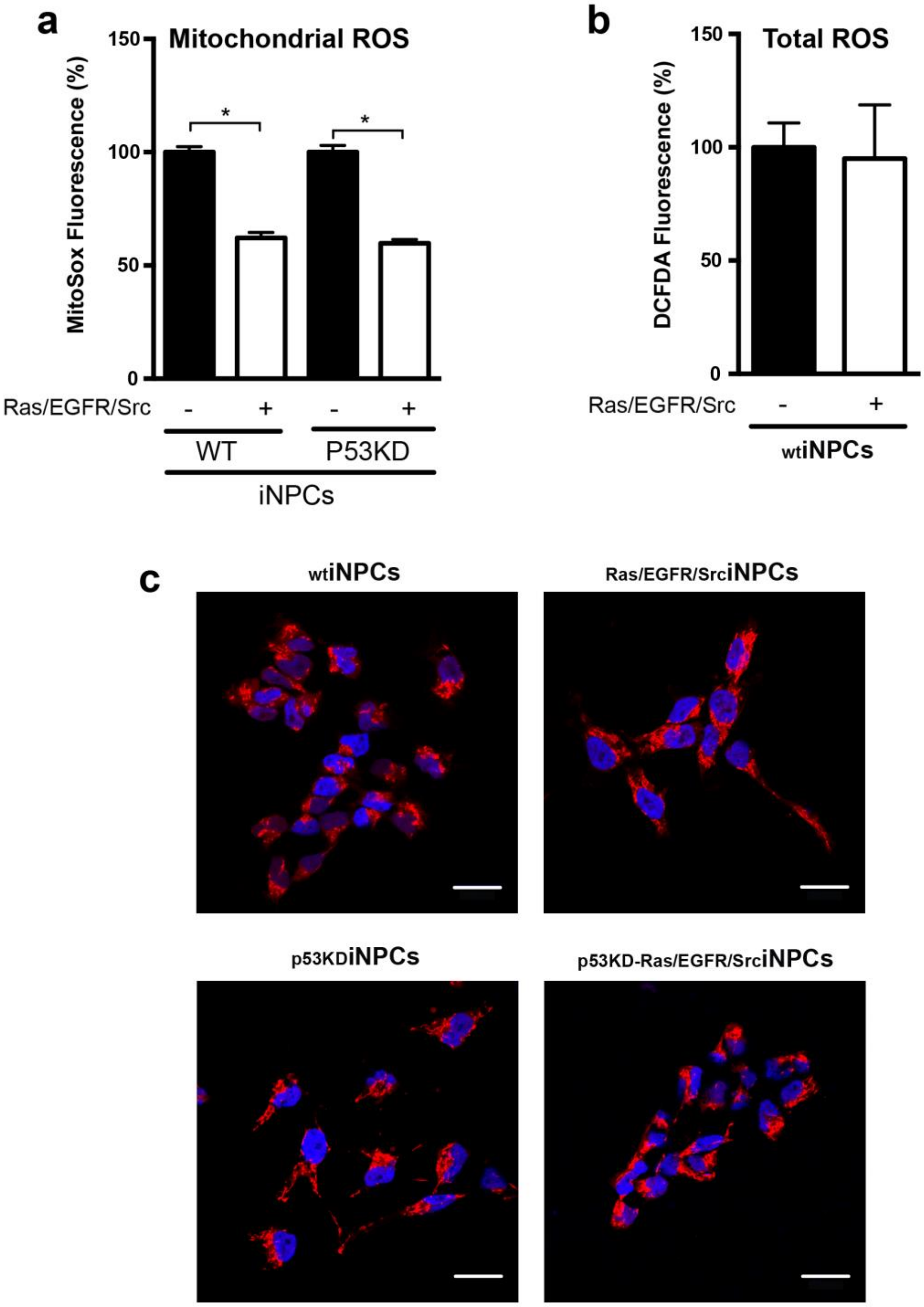

Sancho-Martinez *et al.* Supplementary Figure 4

Supplementary Figure 4. **Transformation of iNPCs induces mitochondrial-associated changes**

a) Mitochondrial ROS production was measured by flow cytometry with MitoSox and revealed that transformation of iNPCs affecting the PI3K/MAK pathways results in increased buffering of mitochondrial superoxide radicals (n=3/group with technical duplicates). **b)** Total ROS levels were measured by flow cytometry with H_2DCFDA staining. No differences in total ROS production are observed when comparing transformed and wild-type iNPCs (n=3/group with technical duplicates). **c)** Representative pictures indicating that transformation leads to mitochondrial network fragmentation in human iPSC-derived iNPCs as indicated by Mitotracker analysis (n=3/group with technical duplicates). Data are represented as mean +/- SD. *p*-values were calculated by Student's *t*-test. *p<0.05. Scale bars: 20μm (c).

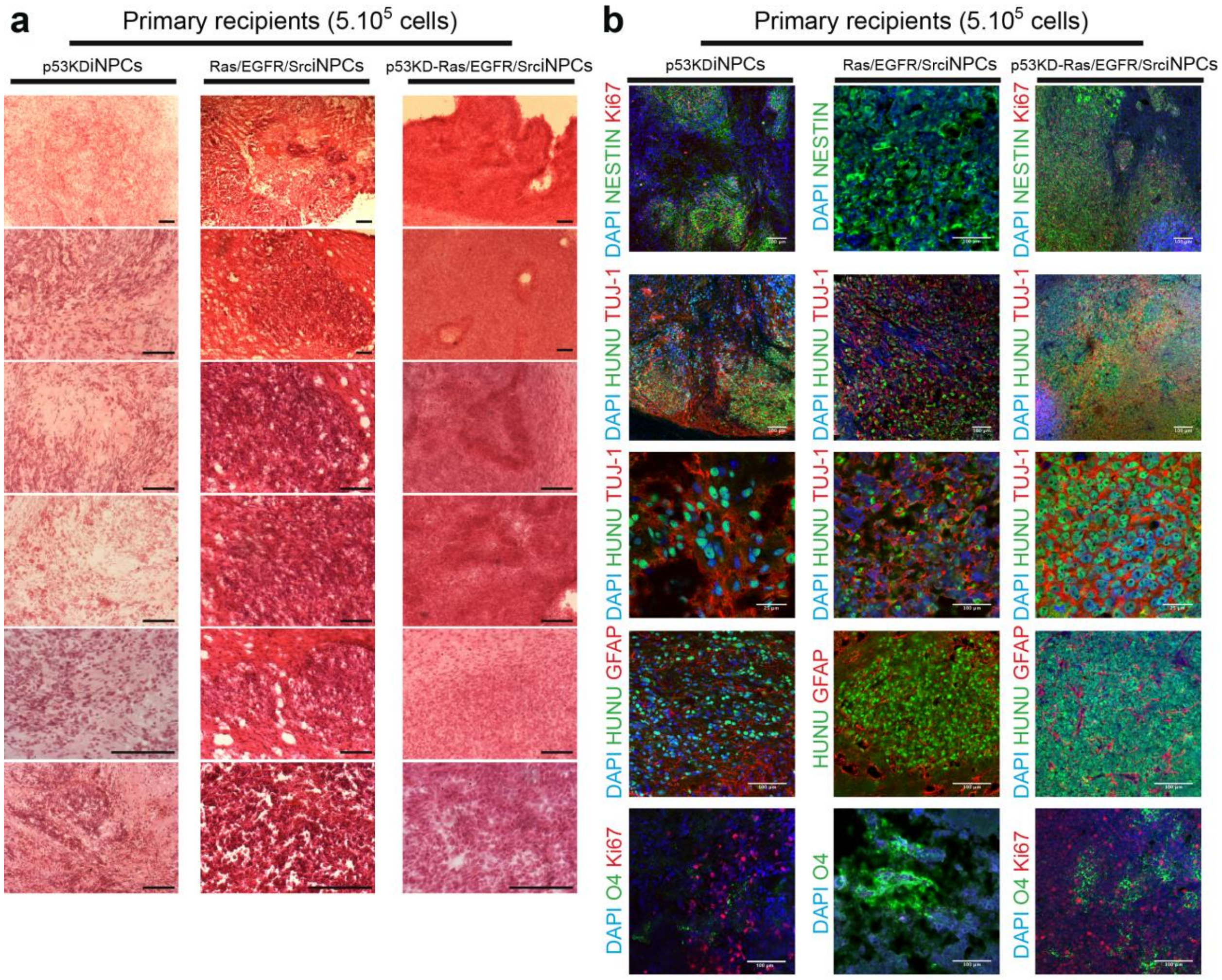

Sancho-Martinez *et al.* Supplementary Figure 5

Supplementary Figure 5. Transformation of **human iNPCs results in the formation of brain tumors containing differentiated derivatives and undifferentiated stem cell populations**

a) Hematoxylin-Eosin staining demonstrating the presence of highly aggressive brain tumors upon xenograft transplantation into the murine brain. **b)** Immunofluorescence analysis demonstrating the presence of undifferentiated NESTIN+ GTICs as well as differentiated cells belonging to the three major neural lineages upon transplantation of transformed human iPSC-derived iNPCs. In a and b, pictures are representative of primary recipient mice that have received an injection of 5.10^5 cells of the indicated groups. HUNU indicates Human Nuclear antigen staining; O4 indicates oligodendrocyte differentiation; Tuj1 indicates neuronal differentiation; GFAP indicates glioma stem cells and glial differentiation. Scale bars: 200μm (a) ; 100μm, 50μm or 25μm as indicated (b). n=5 animals/group.

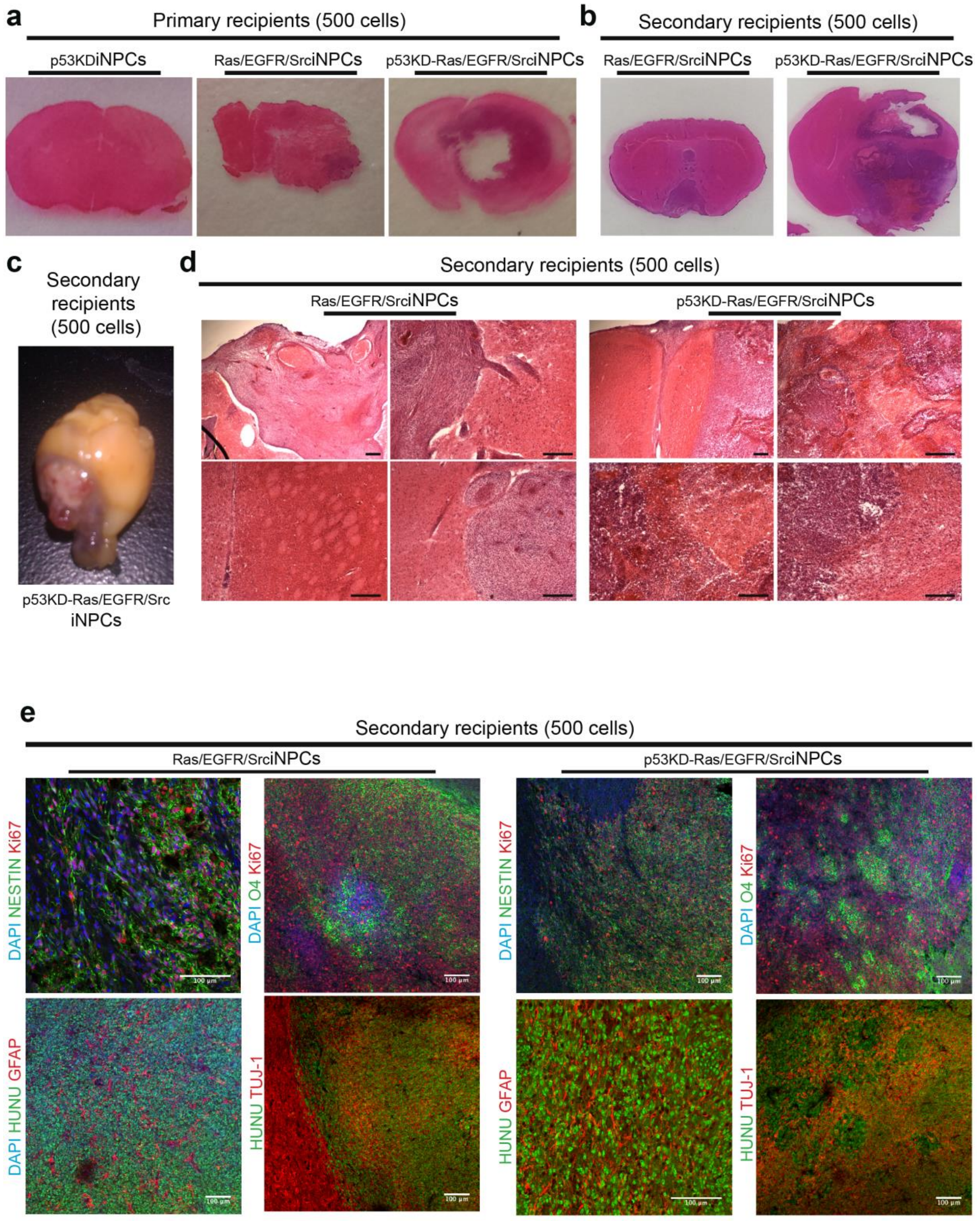

Sancho-Martinez *et al.* Supplementary Figure 6

Supplementary Figure 6. **Limited dilution and serial transplantation experiments transplantation demonstrates the acquisition of GTIC-like properties upon transformation of human iNPCs**

a) Hematoxylin-Eosin staining demonstrating the absence of tumors ($_{p53KD}$iNPCs, 12 months post-injection) or the presence of highly aggressive brain tumors ($_{Ras/EGFR/Src}$iNPCs and $_{p53KD-Ras/EGFR/Src}$iNPCs) upon xenograft transplantation of 500 cells into primary recipients. **b-d**) in b and d, Hematoxylin-Eosin staining demonstrating the presence of highly aggressive brain tumors ($_{Ras/EGFR/Src}$iNPCs and $_{p53KD-Ras/EGFR/Src}$iNPCs) upon xenograft transplantation of 500 cells into secondary recipients. In c, photograph of a brain harvested 95 days after receiving 500 $_{p53KD-Ras/EGFR/Src}$iNPCs upon secondary transplantation. **e)** Immunofluorescence analysis demonstrating the presence of undifferentiated NESTIN+ GTICs as well as their differentiated derivatives upon serial transplantation of 500 cells. HUNU indicates Human Nuclear antigen staining; O4 indicates oligodendrocyte differentiation; Tuj1 indicates neuronal differentiation; GFAP indicates glioma stem cells and glial differentiation. Scale bars: 200μm (D) and 100μm (E). n=5 animals/group.

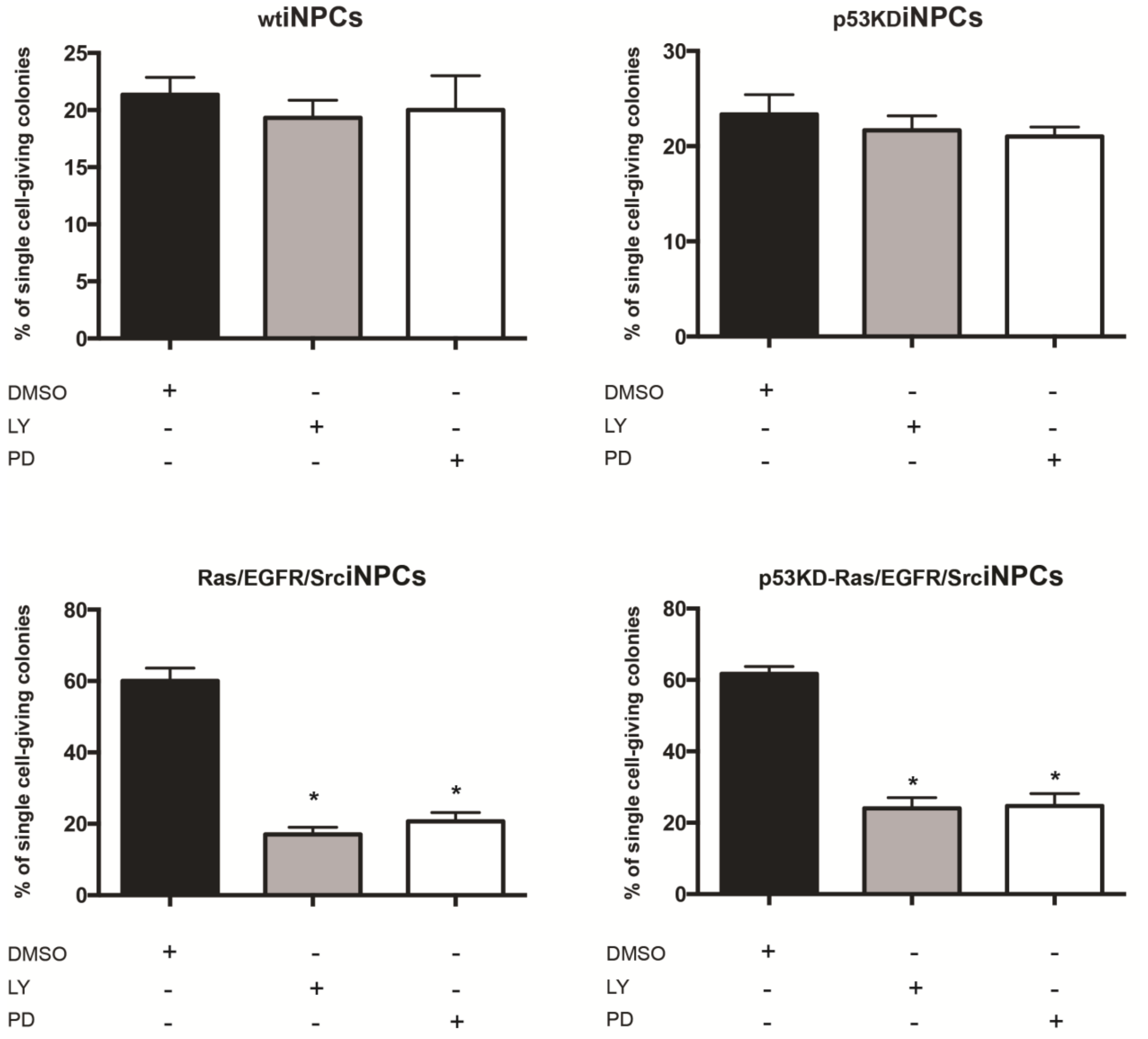

Sancho-Martinez *et al.* Supplementary Figure 7

Supplementary Figure 7. **Aberrant PI3K and MAPK signal are essential for the acquisition of cancer stem cell properties upon transformation of human iNPCs** PI3K and MAPK, and not p53, signalling dysregulation confer stem cell self-renewal properties whereas inhibition of PI3K and MAPK signalling compromises single-cell self-renewal properties. Data are represented as mean +/- SD. *p*-values were calculated by Student's *t*-test. *$p<0.05$, (n=3/group with technical duplicates).

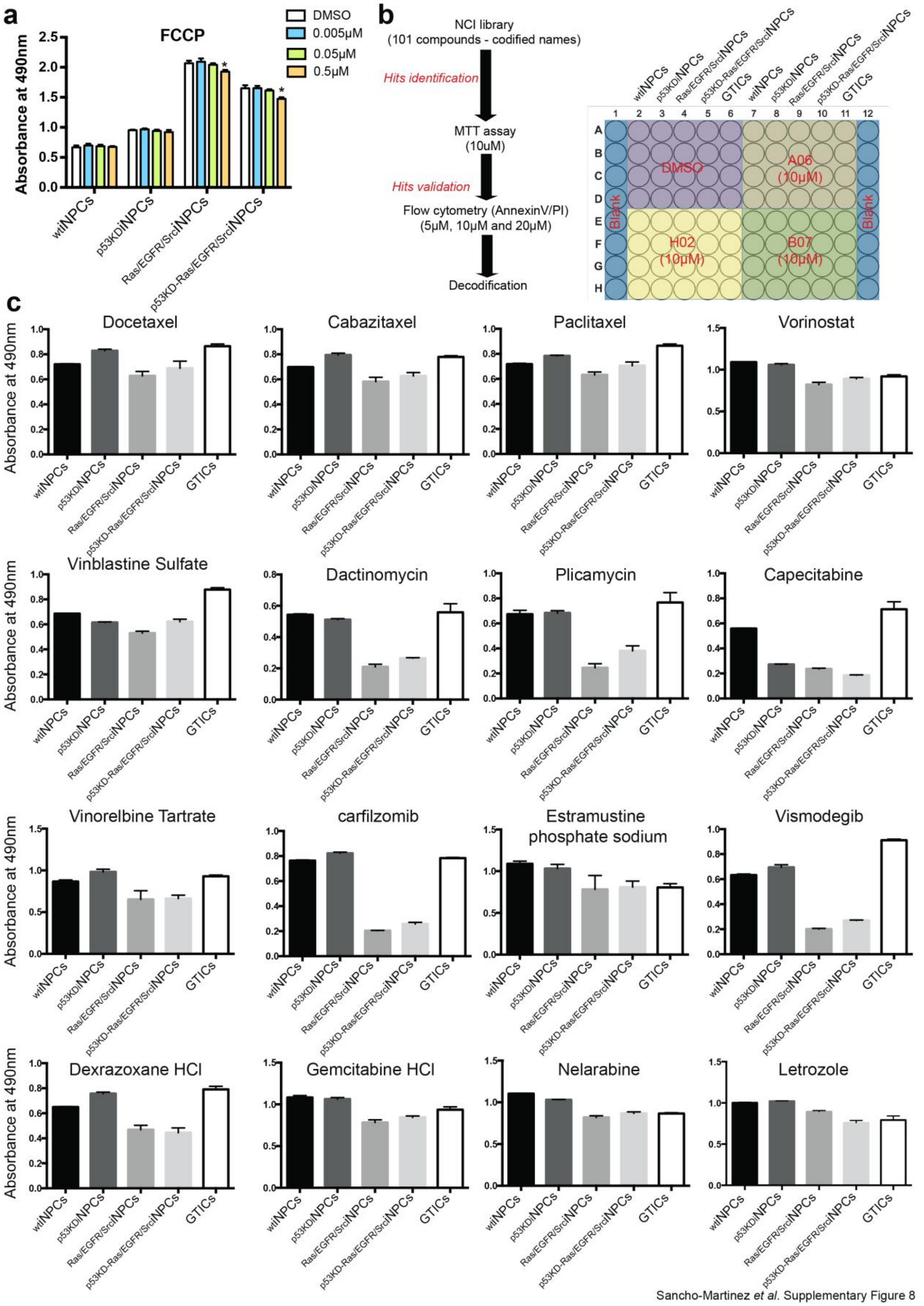

Sancho-Martinez *et al.* Supplementary Figure 8

Supplementary Figure 8. **Application of the generated GTIC-like models for the screening of metabolic modulators and 101 FDA-approved anti-cancer compounds**

a) Bar chart depicting the results of the MTS assay in the presence of the indicated metabolic modulator (FCCP) (n=4 group/condition with 4 technical replicates). **b)** Schematic representation of the workflow used for the screening of compounds (left) and representative 96 well-plate utilized during blind MTS assays for the screening of 101 FDA-approved anti-cancer compounds (right). **c)** MTS results highlighting the effect of the identified compounds in the indicated groups (n=6/group). Data are represented as mean +/- SD.

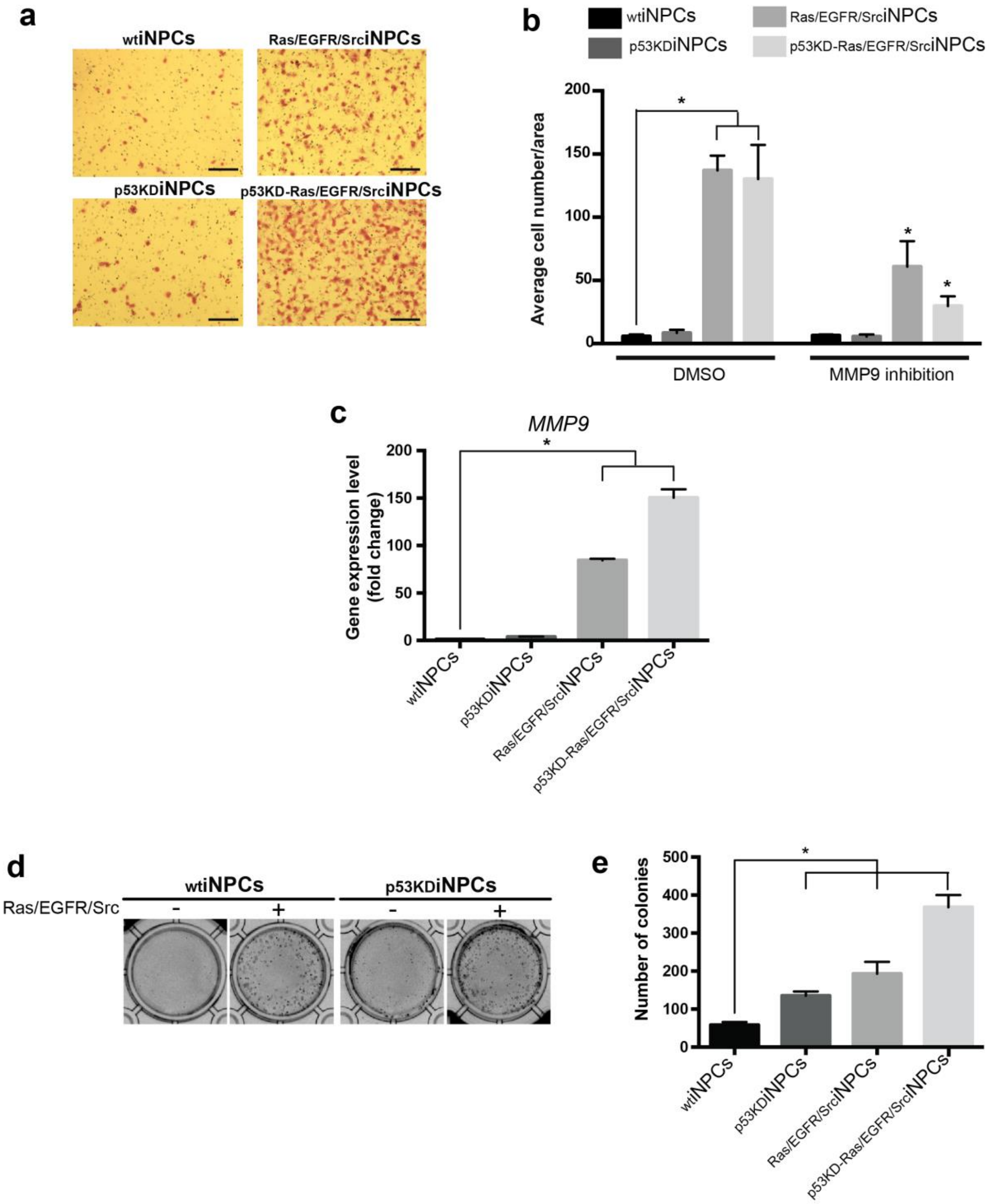

Sancho-Martinez *et al.* Supplementary Figure 9

Supplementary Figure 9. **Aberrant PI3K and MAPK in human iPSC-derived iNPCs leads to the acquisition of GTIC-like properties *in vitro***

a,b) Two-chamber migration assays demonstrates an enhanced migratory phenotype in human iPSC-derived iNPCs upon hyperactivating PI3K and MAPK signaling by overexpression of oncogenic *Ras/EGFR/Src* mutant genes. Representative pictures of the migrated cells (a). Quantification of migrated cells for the indicated lines (b) (n=3/group with technical triplicates). Please note that MMP9 inhibition compromises migration of transformed iNPCs. **c)** Transformation of iPSC-derived iNPCs results in the significant upregulation of *MMP9*, a metalloproteinase involved in glioma cell infiltration (n=3/group with technical triplicates). **d,e)** Transformation of human iNPCs leads to increased self-renewal properties in clonogenic sphere assays. Representative pictures (d) and bar chart (e) depicting the number of colonies for the indicated lines (n=3/group with technical triplicates). Data are represented as mean +/- SD. *p*-values were calculated by Student's *t*-test. *$p<0.05$. Scale bars: 100μm (a).

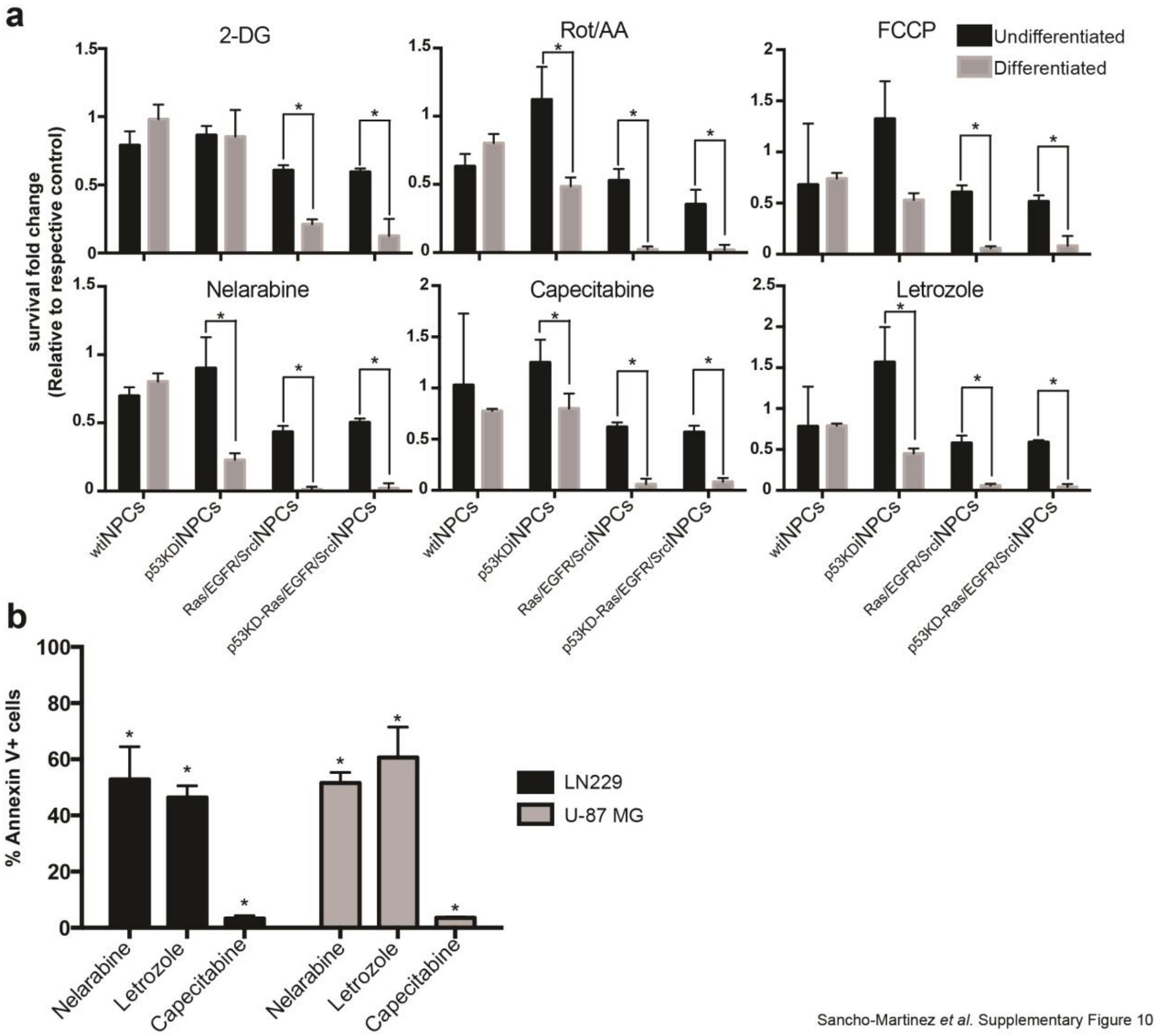

Supplementary Figure 10. **Differentiated transformed iNPC derivatives are more sensitive to cell death**

a) Single-cell differentiation into neuronal lineages significantly enhances response to chemotherapy as compared to undifferentiated iNPCs, only in transformed groups (n=3/condition with technical triplicates). **b)** Validation of the identified compounds in established glioma lines demonstrates significant cell death with capecitabine eliminating most of the plated cells as demonstrated by Annexin-V flow cytometry analysis (n=3/condition with technical triplicates). Data are represented as mean +/- SD. *p*-values were calculated by Student's *t*-test. *$p<0.05$.

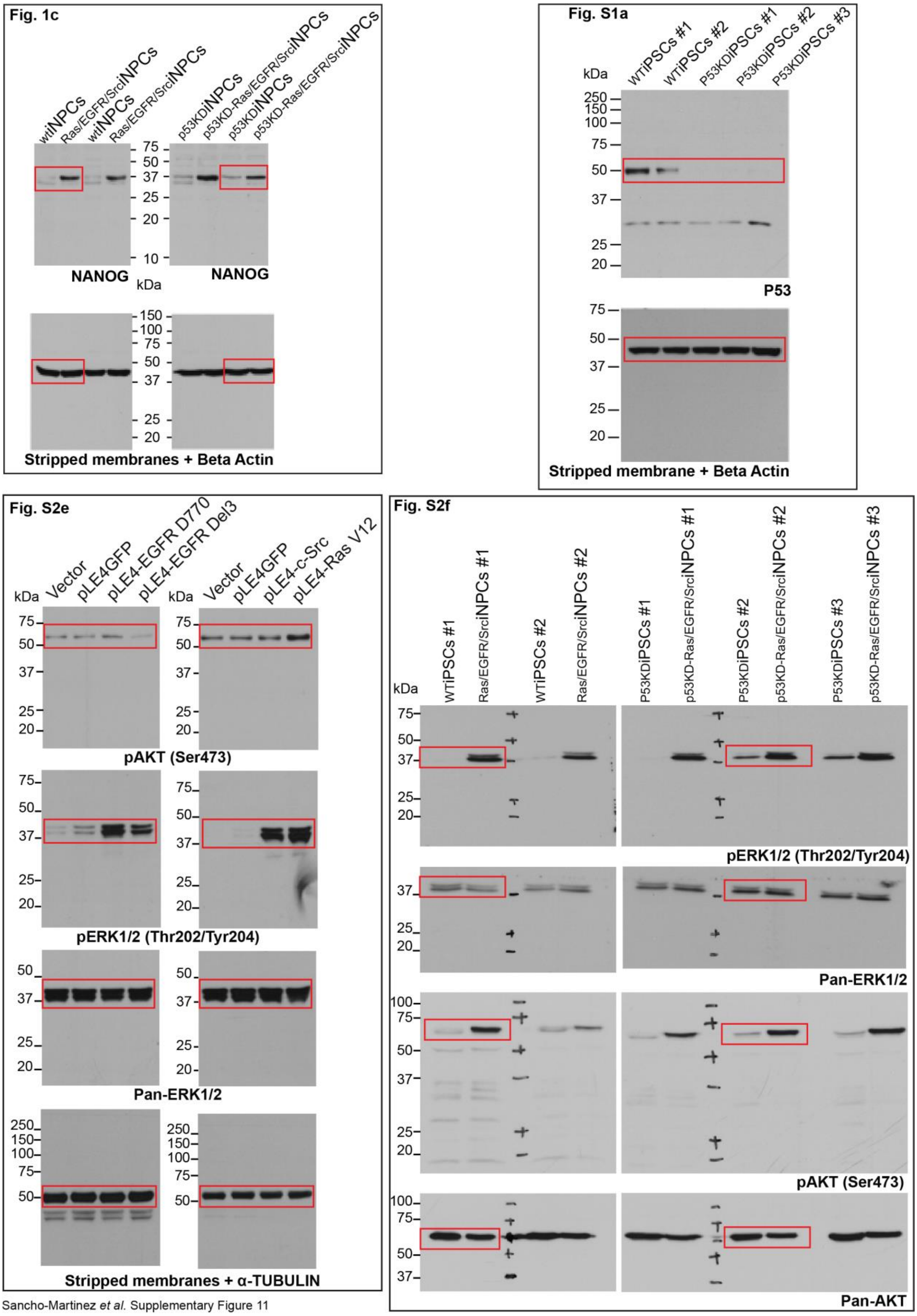

Supplementary Figure 11. **Uncropped images of blots.** Red boxes indicate the cropped regions. Molecular weight markers are indicated in kDa

Supplemental Tables

Supplementary Table 1. **Hypo- and hyper-methylated genes list in primary GTICs, wild-type and transformed iNPCs**

**Hypermethylated in primary GSCs, $_{wt}$NPCs, $_{p53KD-Ras/EGFR/Srci}$NPCs and $_{Ras/EGFR/Srci}$NPCs**					
**ProbeID**	**Gene**	**p53KD-Ras/EGFR/SrciNPCs**	**GSC**	**$_{wt}$NPCs**	**Ras/EGFR/SrciNPCs**
cg14059126	ARL4C	0.897281	0.891716	0.021778	0.892057
cg15918160	AX748201	0.915269	0.93178	0.026541	0.926368
cg05098125	DNAJC24	0.94136	0.969361	0.072046	0.960713
cg23881926	FAM110B	0.942351	0.661806	0.010952	0.925335
cg10177394	LOC100130275	0.736901	0.951588	0.022857	0.973411
cg17754510	LOC100130275	0.930918	0.993085	0.023236	0.989796
cg25460753	LOC100130275	0.805765	0.972128	0.059246	0.973656
cg22840361	NR2F2	0.85876	0.957409	0.019179	0.941821
cg00141929	RFX4	0.960452	0.921934	0.057857	0.93292
cg03384318	TCF7L2	0.924418	0.944148	0.075848	0.96657
cg07591090	TCF7L2	0.909459	0.986892	0.063502	0.942028
cg01231125	UBE2E1	0.932976	0.950143	0.049541	0.888425
**Hypomethylated in primary GSCs, $_{wt}$NPCs, $_{p53KD-Ras/EGFR/Srci}$NPCs and $_{Ras/EGFR/Srci}$NPCs**					
**ProbeID**	**Gene**	**p53KD-Ras/EGFR/SrciNPCs**	**GSC**	**$_{wt}$NPCs**	**Ras/EGFR/SrciNPCs**
cg14557288	ADAMTS14	0.0290178	0.0937263	0.961861	0.014473
cg27358154	ADAMTS14	0.021948	0.176642	0.929478	0.014543
cg00322319	AK027541	0.0110391	0.00765671	0.894801	0.006269
cg10525980	AK027541	0.0254112	0.0113997	0.902375	0.045366
cg13475583	BC016972	0.0315842	0.0847572	0.935071	0.061858
cg23910341	CSNK1G3	0.00533899	0.101575	0.932001	0.031469
cg05910443	CUX1	0.0186411	0.0191429	0.930225	0.012091
cg08502652	FRMD1	0.0369474	0.0595708	0.920359	0.044897
cg07382923	GDF10	0.0846372	0.0360204	0.967609	0.032309
cg01417394	INPP4B	0.0672715	0.0246145	0.949635	0.056823
cg04027043	LMO1	0.0387607	0.0254493	0.967885	0.369783
cg01423916	NUP160	0.0278149	0.0907336	0.922682	0.020879
cg25678052	PLEC	0.0409061	0.029679	0.92665	0.105455
cg21445541	POU2F1	0.0104882	0.0152659	0.978231	0.019407
cg03324578	PRSS42	0.095695	0.0361319	0.961197	0.117473
cg06721601	SH2B2	0.0314043	0.0303622	0.943414	0.034974
cg15374435	SPRY2	0.0133924	0.0171257	0.901504	0.065962
cg07436694	U6	0.0715379	0.0505204	0.947487	0.054363
cg02550308	VPS4A	0.104228	0.0196245	0.92263	0.031359

Supplementary Table 2. **Average survival time of primary recipients mice after intracerebral transplantation of wild-type and transformed iNPCs.** Data are represented as mean +/- SD.

**Experimental condition**	**Average survival time (days) +/- SD**
$_{WT}$**iNPCs** (500,000 cells ; n=5)	No symptomatic manifestations of disease at up to 12 months post-transplantation. No tumor was ever detected. (n=5)
$_{p53KD}$**iNPCs** (500,000 cells ; n=5)	138 +/- 22.6 (n=5)
$_{Ras/EGFR/Srci}$**NPCs** (500,000 cells ; n=5)	53.5 +/- 14.8 (n=5)
$_{p53KD\text{-}Ras/EGFR/Srci}$**NPCs** (500,000 cells ; n=5)	50.5 +/- 5.7 (n=5)
$_{WT}$**iNPCs** (500 cells ; n=5)	No symptomatic manifestations of disease at up to 12 months post-transplantation. No tumor was ever detected. (n=5)
$_{p53KD}$**iNPCs** (500 cells ; n=5)	No symptomatic manifestations of disease at up to 12 months post-transplantation. (n=5)
$_{Ras/EGFR/Srci}$**NPCs** (500 cells ; n=5)	115.2 +/- 28.4 (n=5)
$_{p53KD\text{-}Ras/EGFR/Srci}$**NPCs** (500 cells ; n=5)	96.3 +/- 20.8 (n=5)

Supplementary Table 3. **Average survival time of secondary recipients mice after intracerebral transplantation of wild-type and transformed iNPCs.** Data are represented as mean +/- SD.

Experimental condition	Average survival time (days) +/- SD
$_{WT}$**iNPCs** (500,000 cells ; n=5)	No symptomatic manifestations of disease at up to 12 months post-transplantation. No tumor was ever detected. (n=5)
$_{p53KD}$**iNPCs** (500,000 cells ; n=5)	No symptomatic manifestations of disease at up to 12 months post-transplantation. No tumor was ever detected. (n=5)
$_{Ras/EGFR/Srci}$**NPCs** (500,000 cells ; n=5)	28.5 +/- 12.4 (n=5)
$_{p53KD\text{-}Ras/EGFR/Srci}$**NPCs** (500,000 cells ; n=5)	33.1 +/- 9.4 (n=5)
$_{WT}$**iNPCs**(500 cells ; n=5)	No symptomatic manifestations of disease at up to 12 months post-transplantation. No tumor was ever detected. (n=5)
$_{p53KD}$**iNPCs** (500 cells ; n=5)	No symptomatic manifestations of disease at up to 12 months post-transplantation. No tumor was ever detected. (n=5)
$_{Ras/EGFR/Srci}$**NPCs** (500 cells ; n=5)	92.9 +/- 17.1 (n=5)
$_{p53KD\text{-}Ras/EGFR/Srci}$**NPCs** (500 cells ; n=5)	88.7 +/- 28.8 (n=5)

Supplementary Table 4. **List of the 101 FDA-approved compounds. In red, compounds showing an effect in MTS assays.**

**DRUG NAME**	Celecoxib
Hydroxyurea	Sunitinib Malate
Allopurinol	Axitinib
Fluorouracil (5-FU)	Mitoxantrone HCl
Thioguanine	Pemetrexed Disodium
Mercaptopurine	Gefitinib
Mechlorethamine HCl	Vismodegib
Thiotepa	Crizotinib
Aminolevulinic Acid	Methotrexate
Dacarbazine	Quinacrine
Arsenic Trioxide	Topotecan HCl
Temozolomide	Dasatinib
Busulfan	Pazopanib HCl
Altretamine	Imatinib Mesylate
Floxuridine	sorafenib
Methoxsalen	Raloxifene HCl
Lomustine; CCNU	Pralatrexate
Azacitidine	vandetanib
Decitabine	Vemurafenib
Carmustine	Ixabepilone
Cyclophosphamide	Romidepsin
Uracil mustard	Daunorubicin HCl
Cytarabine; Ara-C	Doxorubicin HCl
Thalidomide	Etoposide
Procarbazine HCl	Tamoxifen Citrate
Streptozocin	Lapatinib Ditosylate
Cladribine	Irinotecan HCl
Ifosfamide	Fulvestrant
Cisplatin	Teniposide
Tretinoin	valrubicin
Dexrazoxane HCl	Docetaxel
Pentostatin	Cabazitaxel
Gemcitabine HCl	Paclitaxel
Nelarabine	Vinblastine Sulfate
Vorinostat	Vincristine Sulfate
Exemestane	Sirolimus (Rapamycin)
Anastrozole	Everolimus
Letrozole	Dactinomycin
Lenalidomide	Plicamycin
Chlorambucil	Bleomycin Sulfate
Mitomycin C	Vinorelbine Tartrate
Mitotane; o;p'-DDD	carfilzomib
Clofarabine	Imiquimod
Pipobroman	Triethylenemelamine
Megestrol acetate	Erlotinib HCl
Bendamustine HCl	Amifostine
Carboplatin	Zoledronic Acid
Oxaliplatin	abiraterone
Fludarabine Phosphate	Melphalan
Bortezomib	Nilotinib
Capecitabine	Estramustine phosphate sodium

Supplementary Table 5. **List of primers used for RT-PCR analysis.**

	**Forward Primer**	**Reverse Primer**
DPPA4	tctggtgtcaggtggtgtgt	tcccttcttgcttttctgga
DNMT3B	cccattcgagtcctgtcatt	ggttccaacagcaatggact
Oct4	gggtttttgggattaagttcttca	gcccccaccctttgtgtt
NANOG	acaactggccgaagaatagca	ggttcccagtcgggttcac
REX1	agaaacgggcaaagacaagac	gctgacaggttctatttccgc
TUJ1	ggccaagggtcactacacg	gcagtcgcagttttcacactc
TBX5	ggagctgcacagaatgtcaa	tgctgaaaggactgtggttg
MEF2C	tgatcagcaggcaaagattg	agtgagctgacagggttgct
MMP9	catcgtcatccagtttggtg	agggaccacaactcgtcatc
GAPDH	agcaatgcctcctgcaccaccacc	Ccggaggggccatccacagtct
Actin	catgtacgttgctatccaggc	ctccttaatgtcacgcacgat
GLUT3	agctctctgggatcaatgctgtgt	atggtggcatagatgggctcttga
HK1	ctgaatagcacctgcgatga	acattcagacggtccagtcc
LDHB	acattcagacggtccagtcc	acattcagacggtccagtcc
PKM2	acattcagacggtccagtcc	gaagatgccacggtacaggt
ALDOA	tgctactaccagcaccatgc	atgctcccagtggactcatc
SDHC	gatggagcggttctggaata	agagagacccctgcactcaa
